# Atomic Defects in Layered Transition Metal Dichalcogenides for Sustainable Energy Storage and the Intelligent Trends in Data Analytics

**DOI:** 10.1002/advs.202521502

**Published:** 2026-01-20

**Authors:** Zheng Luo, Ying Yang, Zizhen Fu, Fengqi Liu, Susu Fang, Kele Xu, Shanshan Wang

**Affiliations:** ^1^ College of Aerospace Science and Engineering National University of Defense Technology Changsha China; ^2^ School of Computer State Key Laboratory of Complex & Critical Software Environment National University of Defense Technology Changsha China; ^3^ School of Advanced Materials Peking University Shenzhen Graduate School Shenzhen Guangdong China

**Keywords:** atomic defects, machine learning, sustainable energy storage, TEM analytics, transition‐metal dichalcogenides

## Abstract

Defects in layered transition metal dichalcogenides play a crucial role in the development of high‐energy‐density and high‐safety electrochemical devices for sustainable energy storage systems. Although transmission electron microscopy prevails as an indispensable tool for visualizing defects at the atomic scale, manual analysis in such data‐intensive scenarios generally lacks both efficiency and accuracy. Fortunately, the emergence of machine learning is transforming the paradigm of electron microscopy data analytics, offering a potent tool to expedite the discovery of novel structures and knowledge. In this review, we briefly introduce the atomic structures of typical defect configurations in transition metal dichalcogenides, along with their beneficial effects on electrochemical redox kinetics and stability when used in batteries and supercapacitors. Then, the latest innovations in the defect‐engineered transition metal dichalcogenides for advanced energy storage devices, and the progress made in machine learning methodologies for their application in high‐throughput electron microscopy analytics are systematically summarized. Finally, this review is concluded with perspectives on the remaining challenges and future opportunities in intelligent defect characterizations and engineering toward the next‐generation energy storage systems.

## Graphical abstract

This review comprehensively summarizes the atomic defects in TMDs for their applications in sustainable energy storage devices, along with the latest progress in ML methodologies for high‐throughput TEM data analysis, offering insights on how ML‐empowered microscopy facilitates bridging structure–property correlation and inspires knowledge for precise defect engineering.

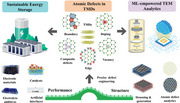

## 1 Introduction

**FIGURE 1:**
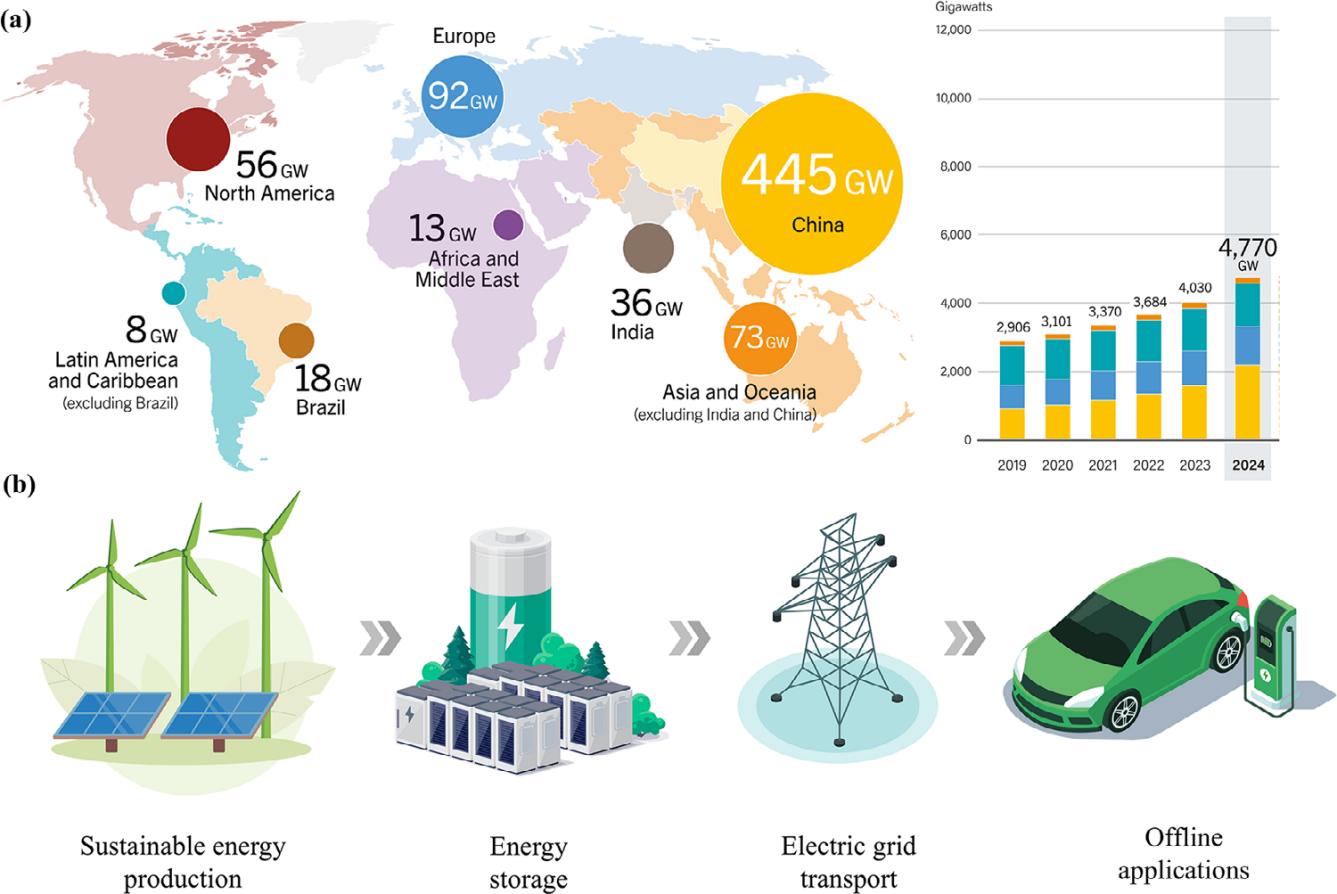
The full chain of a sustainable energy system, along with the recent statistical data on its installed capacity. (a) Statistical data on the installed capacity of sustainable energy in 2024. Copyright 2025, REN 21 [2]. (b) Schematic showing the full chain of a sustainable energy system.

The global upsurge in energy demand and environmental crisis has compelled a shift in the energy consumption structure from fossil fuels to clean and sustainable energy sources [1]. Driven by technological advancements in collecting solar, wind, and hydropower energy, etc., the renewable power capacity has reached a record high of approximately 4770 GW with a remarkable increase of 741 GW over the past year (Figure 1a), accounting for 13.5% of total final energy consumption [2]. Typically, energy storage devices, such as pumped storage hydropower, electrochemical energy storage, and compressed air energy storage, are indispensable in the entire chain of sustainable energy systems, which can convert intermittent energy into stable forms to ensure consistent outputs when required [3]. Among these, electrochemical energy storage devices, including batteries and supercapacitors, have demonstrated unparalleled advantages in terms of energy density, lifespan, response speed, and flexibility, exhibiting annual growth in the proportion of the energy storage market [4, 5]. Moreover, the prosperity of electrochemical energy storage devices in the terminal applications, like electric vehicles and portable electronic devices, facilitates the establishment of an eco‐friendly chain to further curtail fossil fuel usage and address environmental degradation (Figure 1b) [6, 7]. Currently, lithium‐ion batteries (LIBs) represent the most developed electrochemical energy storage technology, and occupy the leading position in the consumption markets [8, 9]. However, the state‐of‐the‐art LIBs, utilizing graphite as the anode and a nonaqueous solution as the electrolyte, are nearly reaching their theoretical limits, but still fall short of the ever‐growing energy storage demands [10, 11]. More importantly, there have been incessant reports of fires and explosions related to LIBs, which raise intensive safety concerns for large‐scale energy storage [12]. To concurrently improve the energy density, lifespan, and safety of LIBs, it is highly expected to explore innovative electrode materials, or even investigate alternative battery systems with an inherently nonflammable nature, like solid‐state batteries [13], aqueous metal‐ion batteries [14], and flow batteries [15].

Layered transition metal dichalcogenides (TMDs), primarily with a formula of MX_2_ (M = Mo, Ti, W, Re; X = S, Se, Te), have garnered significant scientific attention in the fields of energy storage, conversion, and electronic devices, owing to their tunable physical, chemical, and electronic properties [16, 17, 18]. Particularly, interlayers in these materials are bound together by a weak van der Waals (vdWs) force similar to that of graphite, providing effective channels for the reversible intercalation/deintercalation of alkali metal ions or even multivalent ions [19]. Based on the intercalation chemistry, the first‐generation Li‐based batteries were successfully developed by utilizing TiS_2_ and MoS_2_ as cathodic hosts for Li^+^ storage, delivering an energy density of 60–65 Wh kg^−1^. Furthermore, discharging the battery at the anode side to a deeper extent can initiate a conversion reaction with the formation of M and A_2_X (where A = Li, Na, K), potentially elevating the energy density of battery systems to an unprecedented level [20, 21]. In addition, the exceptional specific surface area and in‐plane strength naturally position TMDs as promising candidates for use as catalysts, additives, and protective layers in certain specialized applications, such as catalyzing the sulfur redox chemistry in Li–S batteries and optimizing the solvation environment in polymer electrolytes [22]. However, pristine TMDs inherently possess several drawbacks when directly employed in energy storage devices, including limited active sites, low electrical conductivity, and compromised structural stability during repeated cycling processes, which greatly hamper their practical applications [23, 24].

According to the second law of thermodynamics, defects are inevitable in crystalline materials, which break the local periodicity of a perfect lattice with tailored electronic structures and properties. Prior research has shown that rationally designing defects in TMDs can effectively optimize carrier concentration and the band structure to enhance the electrical conductivity [25]. Therefore, various defect engineering strategies have been adopted to boost surface chemical activity and decrease the reaction energy barrier for rapid redox kinetics [26, 27]. Typically, revealing the defect structures at atomic resolution is the cornerstone to establishing structure–property correlation for precise defect manipulations, which nowadays can be realized by advanced transmission electron microscopy (TEM) technologies in picometer‐level precision. By selectively collecting signals from different types of electron beams, TEM can comprehensively reflect the structural information of a material with high spatial resolution from multiple perspectives. For example, high‐resolution transmission electron microscopy (HR‐TEM) collects atomic structure information from the phase contrast among all the parallel diffraction and transmission electron beams, while focusing the electron beam to conduct a point‐by‐point scan of the specimen via scanning transmission electron microscopy (STEM) enables the acquisition of compositional information from each atomic column [28]. However, the prosperity of TEM technologies, on the one hand, produces a large quantity of graphic datasets varying with time, position, or imaging conditions for a comprehensive defect investigation; on the other hand, it poses new challenges for researchers concerning efficiency and accuracy when dealing with such a data‐intensive scenario. Therefore, a high‐throughput analytic approach with promoted accuracy, efficiency, and statistical rigor deserves in‐depth consideration.

**FIGURE 2:**
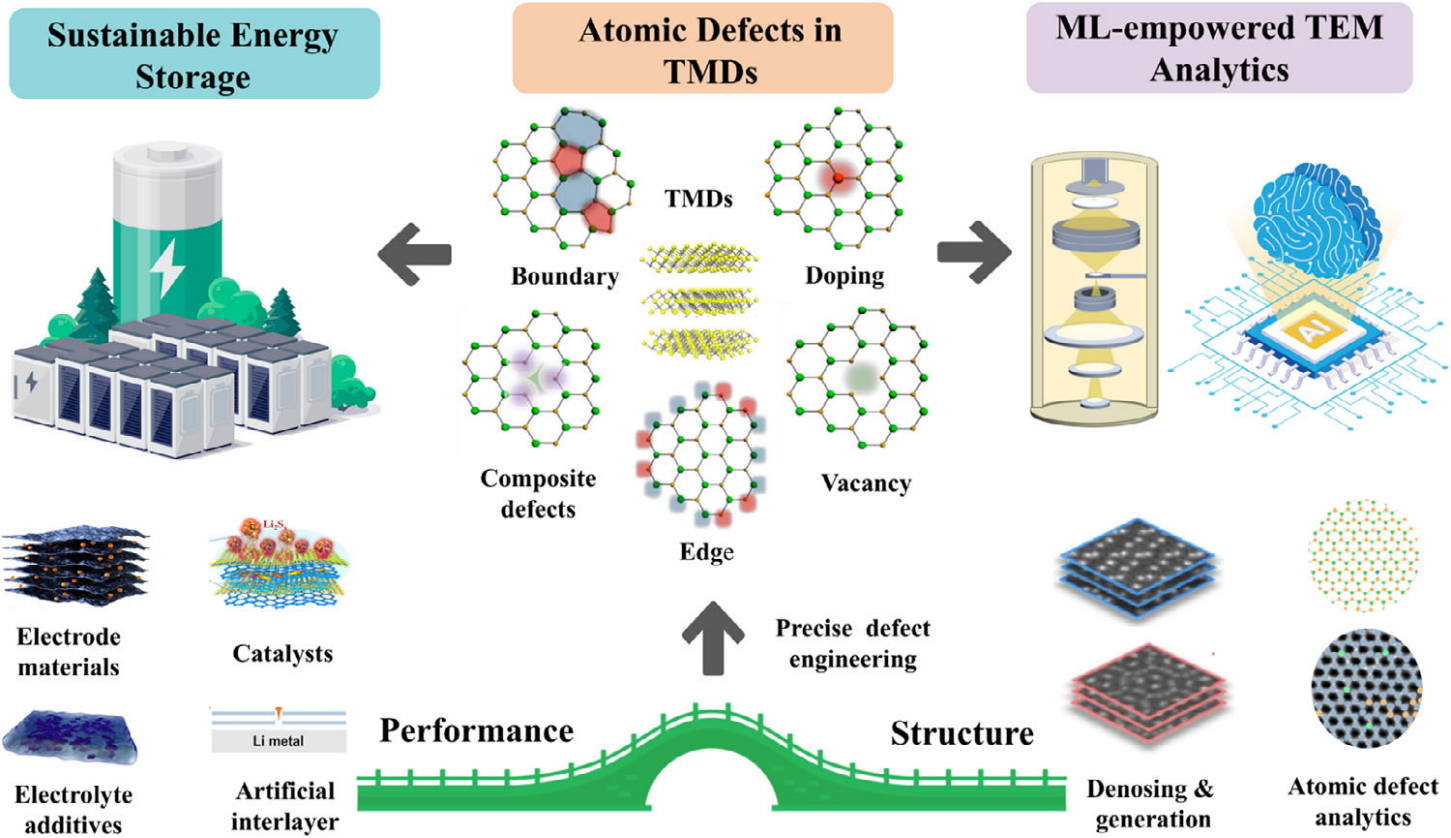
Schematic showing the main contents of this review, including the atomic defects in TMDs, their applications in sustainable energy storage devices, and the ML‐empowered defect analytics.

Fortunately, owing to the exceptional ability to establish relations between images and contents, machine learning (ML) has proven its superiority over human experts in the realm of image processing, thereby showcasing great potential for efficient TEM analytics in various scenarios, such as vacancy identification, phase segmentation, and crystal symmetry determination [29]. Specifically, ML algorithms can be categorized into supervised and unsupervised types, depending on whether pre‐labeled data are required for model training [30, 31]. Furthermore, as a crucial sub‐field of ML, deep learning (DL) with a large depth of the comprised layers in the neural networks can emulate the way the human brain processes data, offering a more efficient and robust tool for addressing complex TEM tasks with larger data volumes [32]. Currently, ML‐based approaches evolve rapidly and have been revolutionizing the paradigm of data analytics in electron microscopy. Herein, beginning with the introductions of atomic defects in TMDs and their effects on property manipulation, the defective TMDs utilized in high‐performance electrochemical energy storage devices, along with the latest progress in ML methodologies for high‐throughput TEM data analysis, are comprehensively elaborated, aiming to offer insights on how ML‐empowered microscopy facilitates bridging structure–property correlation and inspires knowledge for precise defect engineering (Figure 2). Finally, this review is concluded with perspectives on the remaining challenges and future research opportunities for developing next‐generation energy‐related devices in a more efficient and intelligent way.

## 2 Classification and Visualization of Atomic Defects

**FIGURE 3:**
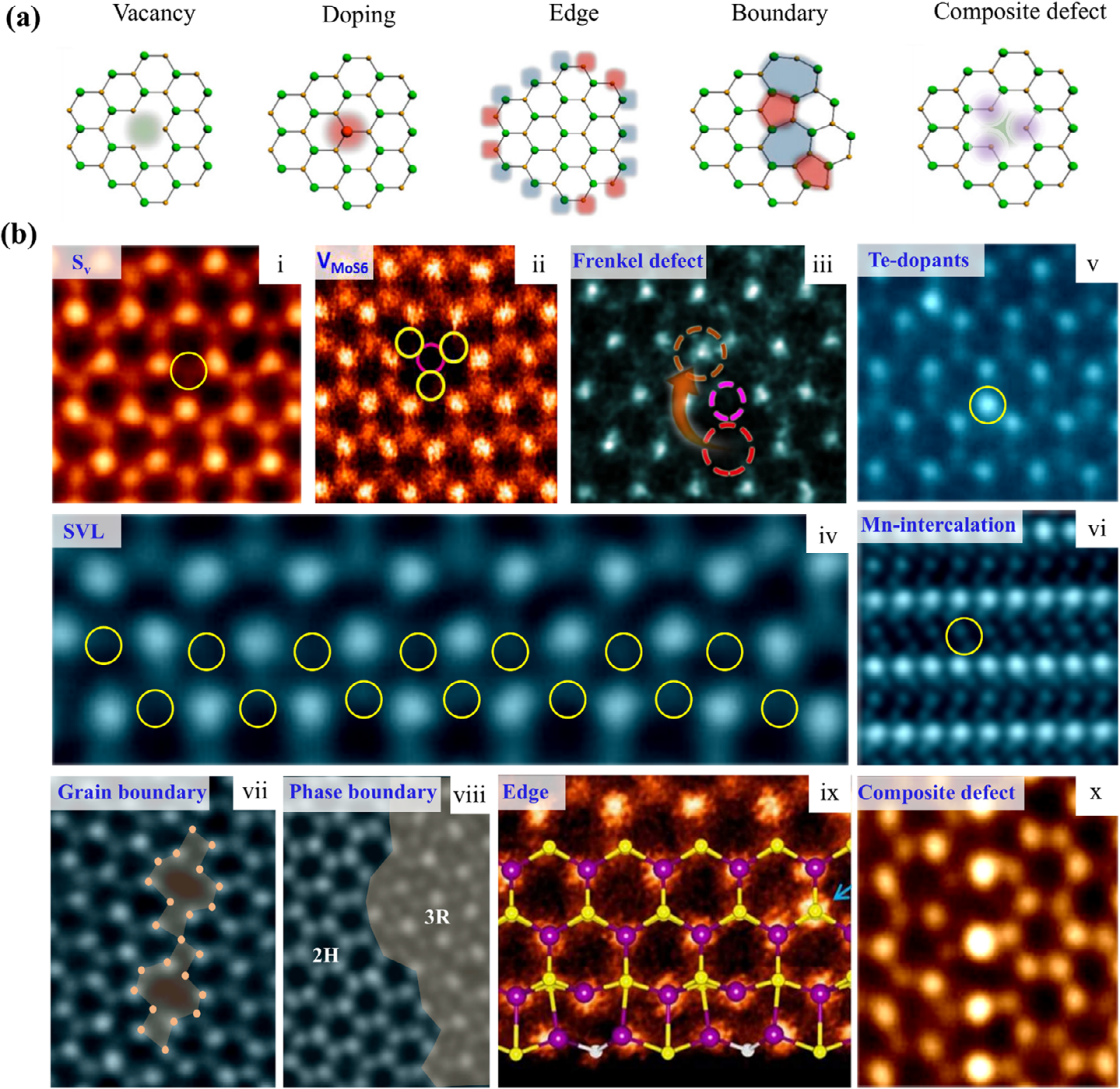
Typical atomic defects in 2D TMDs. (a) Atomic models of various types of defects in 2D TMDs [35]. Copyright 2019, Springer Nature. (b) Atomic‐resolution STEM‐ADF images of different atomic defect types. (i) S vacancies in MoS_2_; (ii) V_MoS6_ in MoS_2_;(iii) Frenkel defects in MoS_2_; (iv) SVL in MoS_2_; (v) Te substitutions in MoS_2_; (vi) Mn intercalation in NbS_2_; (vii) Grain boundaries in MoS_2_; (viii) Phase boundaries between 2H and 3R phase in MoS_2_; (ix) Sulfur vacancies induced reconstruction of the Mo‐terminated edge; (x) Composite defects with a row of Pt single atoms locating at the Mo site of grain boundaries in MoS_2_. Panels 3b(ii) and (ix) are reprinted with permission from ref [37], Copyright 2013, American Chemical Society. Figure 3b(iii) is reprinted with permission from ref [38], Copyright 2022, Springer Nature. Figure 3b(vi) is reprinted with permission from ref [39], Copyright 2024 Springer Nature. The remaining images are cropped from our row datasets.

An ideal single crystal is perfectly arranged. However, in the real world, defects are inevitable for maintaining thermal equilibrium [33]. Defects refer to those structures that break the periodicity and symmetry of a perfect lattice, whose concentration and distribution profoundly influence the chemical and physical properties of TMDs, as well as the consequential electrochemistry performance when employed in energy storage devices [34, 35]. Based on the atomic structures, defects can be classified into five types in TMDs (Figure 3a): (1) vacancies, the absence of atoms in the normal lattice site; (2) heteroatoms dopants, the heteroatoms introduced in the normal lattice; (3) boundaries, the interface between grains or phases; (4) edges, the interface between materials and vacuum; (5) composite defects, the defects that locally contains two or more distinct types of single defects. Currently, TEM techniques provide a powerful platform for precisely visualizing and investigating these defects at the atomic scale, which helps establish the structure–performance relationship for specific defect designs in TMDs rapidly [36]. This section summarizes the atomic structures of different types of defects in TMDs.

### 2.1 Vacancies

Vacancies are referred to those sites with missing lattice atoms, which usually induce lattice distortion and local charge redistribution at the band gap level [40, 41, 42]. From a thermodynamic perspective, vacancies can be divided into intrinsic and extrinsic types depending on the inducing factors. Intrinsic vacancies, like anion vacancies, Schottky defects (the anion–cation vacancy pair), are inevitable within a material due to the local energy fluctuations from the atomically thermal vibrations, whose concentration is only proportional to the temperature [43]. On the contrary, the concentration and distribution of extrinsic vacancies can be finely modulated by the intensity of external stimuli, which drives the original lattice to deviate substantially from its equilibrium state, providing ample space for manipulating many structure‐sensitive properties of TMDs [44, 45, 46]. Typically, both metal and chalcogen atoms can be excited in TMDs, but the loss of metal atoms requires a much higher excitation energy compared to that of displacing a chalcogen atom [47]. Therefore, the most prevalent form of vacancy is the removal of a single chalcogen atom in TMDs, exhibiting a decreased contrast in ADF‐STEM images (Figure 3b(i)) and minimal strain or bond reconstruction in the vicinity of the vacancy site [48]. On the contrary, single metal vacancies are demonstrated energetically unfavorable as their neighboring chalcogen atoms are prone to missing simultaneously, forming MX_x_ clusters instead, like the V_MoS3_, V_MoS6_ (Figure 3b(ii))^[^ ^37^ ^]^ and even Frenkel defects (Figure 3b(iii)) [38] in MoS_2_ if the nearby S vacancies capture the excited Mo atom. Additionally, chalcogen vacancies would be aggregated with the increase in vacancy concentration, forming vacancy lines with flexible lattice distortion (Figure 3b(iv)) [49]. Li et al. have systematically studied the agglomeration process of S vacancies under a moderate acceleration voltage of 80 kV [50], where only single S atoms can be ejected from the MoS_2_ lattice, forming a series of S vacancy lines (SVL) with multiple bond reconstructions. Furthermore, broadening the S vacancy line from 1SVL to 4SVL surprisingly leads to decreased bap gap ranging from semiconductor to conductor based on the DFT calculations, and a novel rectangular MoS structure is created by further broadening the vacancy lines, displaying an obvious anisotropy in the thermal elastic modulus, lattice thermal conductivity, electron thermal conductivity, and electrical conductivity properties.

### 2.2 Heteroatom Dopants

Heteroatom dopants are defined as the heteroatoms that substitute into, adsorb onto, or intercalate within the normal lattices, which can either exist naturally or be deliberately introduced during the synthesis process, thereby significantly altering the skeleton and electron state of TMDs [51]. To ensure successful doping of TMDs, the introduced foreign atoms should be carefully considered, such as atomic radius, electron concentration, valence state, etc., to maintain the intrinsic 2D configurations of TMDs [28]. Regarding the substitutional dopant atoms, elements in the same group of the periodic table with the similar radius, valence and coordination manner are generally suggested, which have been extensively demonstrated to form stable and continuous doped TMDs, like Se‐doped VS_2_ [52], Mo‐doped WS_2_ [53], and Te‐doped MoS_2_ (Figure 3b(v)) [54], with the transition metal or the chalcogen atoms being substituted. On the contrary, for those dopant atoms whose atomic radius or coordination manner cannot match with the host 2D TMD crystals, like Pt [55], Co [56], Au [57], etc., they tend to locate on the surface of TMDs rather than being incorporated in the lattice, resulting in lattice strain and out‐of‐plane distortion. In most cases, adsorbents prefer to locate on the chalcogen vacancy sites on the clean surface of monolayer TMDs, or aggregate into clusters depending on their surface migration capability [58]. Additionally, TMDs with interlayer vdWs gaps enable the intercalation of foreign atoms driven by electric or chemical potential, which not only serves as the foundation for electrochemical energy storage of TMDs but also introduces an additional dimension for structural and property manipulations [59]. For example, Gong et al. successfully achieved Sn intercalation into vdWs gaps of TMDs via a solution method to facilitate the conversion efficiency of lithium polysulfides in a Li–S battery [60]. They also obtained a series of intercalated 2D TMDs, like V_x_NbS_2_, Cr_x_NbS_2_, Mn_x_NbS_2_ (Figure 3b(vi)), Fe_x_NbS_2_, Co_x_NbS_2_, Co_x_NbSe_2_, Fe_x_TaS_2_ via a flux‐assisted growth method [39], showing distinctive magneto‐transport properties. However, the design principle regarding the compatibility between the intercalated guest atoms and the host substrate still awaits further in‐depth investigation.

### 2.3 Boundaries

Boundaries refer to the interfaces between grains or phases, which induce a certain lattice mismatch, and have a profound impact on the local band structure and charge transfer behavior of the 2D TMDs. Generally, grain boundaries (GBs) originate from the atomic stitching between two grains with different lattice orientations, which are observed to be formed by the dislocation cores that are spaced apart [61]. Similar to graphene, hexagonal TMDs consist of a variety of dislocation core types like 5‐ and 7‐fold (5|7) rings, 4|8 rings (Figure 3b(vii)), 6|8 rings, but with slightly varied substitution arrangement between metal and chalcogen atoms [62]. For example, He et al. obtained wafer‐scale MoS_2_ nanosheets with high GB density (∼10^12^ cm^−2^) as high‐efficiency catalysts, where the HAADF‐STEM image demonstrates the combination of 5|7 and 6|8 rings at the GBs [63]. Additionally, the structural diversity of GBs is more obvious in low‐symmetry TMDs, like the 1T’‐ReS_2_ with two reverse vertical orientations, namely “face‐up” and “face‐down,” which gives rise to the formation of multiple new types of GBs with different orientation mismatches [64]. Wang et al. also observed a series of overlapping GBs in 1T’‐ReS_2_ with tuned band structures [65]. A phase boundary is a specific type of boundary in TMDs. As demonstrated, most monolayer TMDs possess two or more types of phase structures, like the 1H phase and 1T phase in monolayer MoS_2_ [66]. Also, the types of crystal phases can be further enriched in multilayer TMDs due to the existence of numerous possible stacking configurations. The two most common and low‐energy stacking configurations are the 2H (AA) and the 3R (AB) types (Figure 3b(viii)) [67]. Compared to grain boundaries, more pronounced band bending and local charge accumulation can be generated at the phase boundaries due to the variance in band structure between two distinct phases, which also outperforms the electrochemical performance of each individual phase [68].

### 2.4 Edges

The termination of domains brings uncoordinated atoms hanging in the vacuum with varied geometry, showing distinctive properties compared to the basal plane of TMDs. In hexagonal TMD materials, two kinds of edge structures, including armchair edge and zigzag edge, have been observed [69], where the dominance of zigzag edge confirms its energetic favorability [70]. Besides, DFT calculations prove the semiconductive feature of the armchair edge and the metallic feature of the zigzag edge, which is beneficial for the charge transfer when employed in electrode materials and catalysts. Typically, the zigzag edges exhibit two potential termination directions, namely chalcogen and metal, which also possess different activity and stability characteristics. For example, the Mo‐terminated edge is theoretically proven to be electrochemically active, while the S‐terminated edge is as inert as the basal plane of MoS_2_ [71]. But fortunately, the S‐terminated edge can be converted to the Mo‐terminated edge at high temperature from the S depletion [72]. In addition, Zhou et al. found a new type of edges containing 50% of S vacancies with obvious bond reconstruction in MoS_2_ (Figure 3b(ix)), which can inherit the metallic feature of the ideal Mo‐terminated edges [37]. Three approaches are commonly employed to enrich the edges in TMDs: building dendritic structures, creating nano‐pores, and generating nano‐cracks, which have been effectively utilized to boost the electrochemical performance for energy storage devices [73, 74].

### 2.5 Composite Defects

In practical situations, defects do not exist in a single form, but in a combination of multiple types of defects, which gives rise to manipulating the properties of TMDs over a wider range. For example, Warner et al. have observed a row of Pt single atoms that substitutes the Mo atoms on the grain boundary of MoS_2_ (Figure 3b(x)), showing an obviously decreased band gap compared to each single defect type [75]. Fan et al. theoretically and experimentally demonstrated a series of foreign substitutions surrounding the S vacancy center, where the triangular‐shaped Co atom cluster centered by a S vacancy is found to greatly alter the local charge distribution [76]. Also, Ding et al. introduced N atoms to activate the S‐terminated edges for high‐efficiency catalysis [77]. Despite these contributions, the design principle and precise creation of these composite defects still leave a huge space yet to be explored.

Except for the abovementioned defects, there are other types of defects in TMDs, such as stacking faults, cracks, and holes, etc., as the atomic structures continue to evolve [28]. Furthermore, the concentration and distribution of these defects have also been demonstrated to significantly impact the physicochemical properties and stability of TMDs. Therefore, the structural diversity of defects in TMDs provides a vast space for property manipulations in energy storage devices

## 3 Defective TMDs for Electrochemical Energy Storage Devices

Batteries and supercapacitors are the two mainstream electrochemical devices for sustainable energy storage [78]. Structurally, they share similarities, both consisting of electrodes and electrolytes to enable the conversion of energy between chemical and electrical forms [79]. To break the current bottlenecks of batteries and supercapacitors in terms of energy density and lifespan, 2D TMDs with large specific area and tunable electronic structures have been extensively investigated as potential electrode materials or catalysts in energy storage devices. However, solely employing raw TMDs in terminal devices encounters several challenges. For example, when utilized as electrode materials, the majority of TMDs can hardly approach their theoretical values under practical conditions due to the semiconductive feature and limited active sites. Moreover, the structural stability of TMDs requires further optimization to endure the repetitive ion intercalation/deintercalation process. Fortunately, the advances of nanotechnology bring opportunities to precisely introduce defects at the atomic level for property manipulation [34]. This section systematically discusses the major effects of defects and reviews the recent progress of defect engineering on 2D TMDs for high‐performance electrochemical energy storage devices.

### 3.1 Effects of Defects in TMDs on Property Manipulation

**FIGURE 4:**
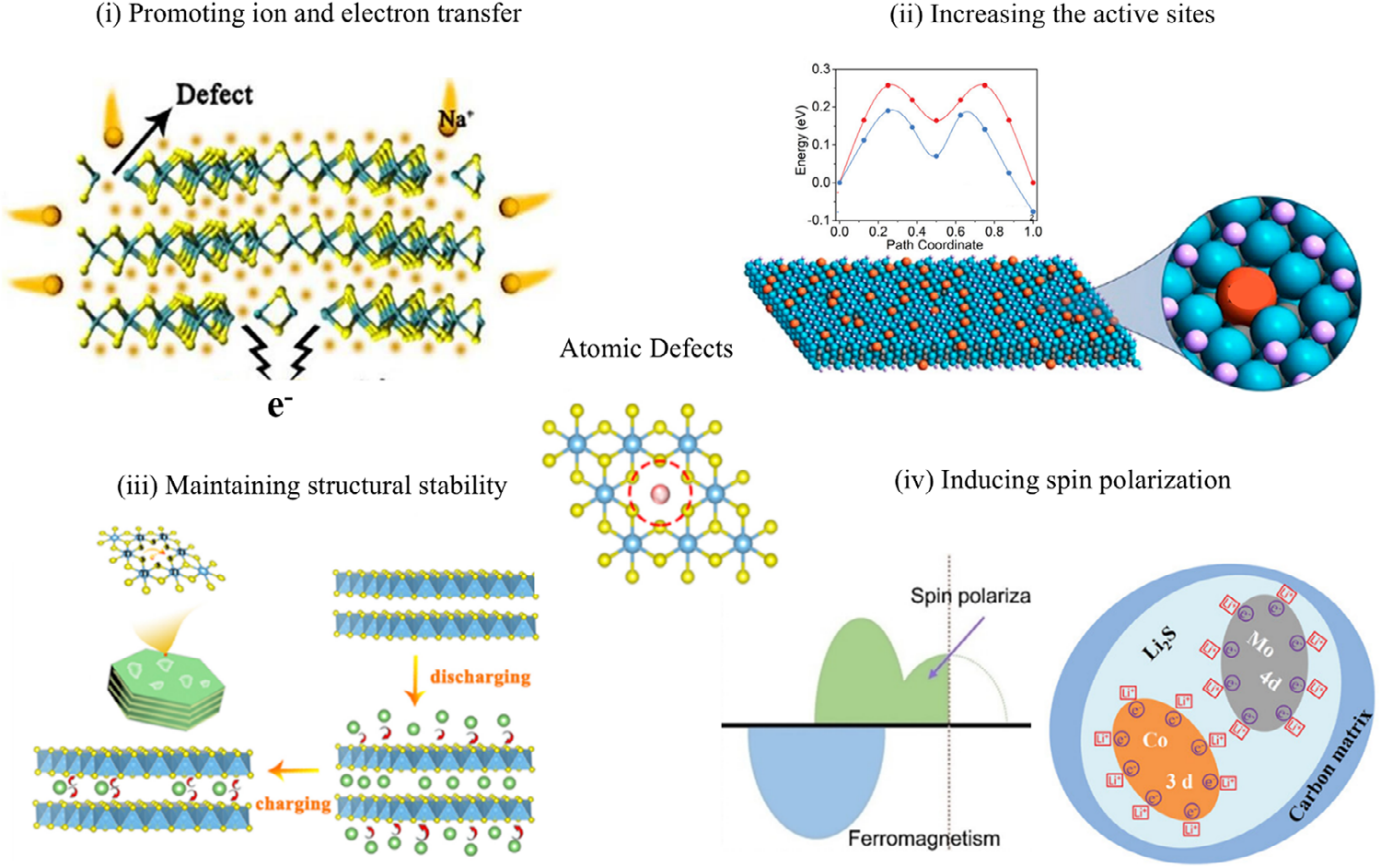
Schematics showing the major effects of defects on the electrochemical performance of 2D TMDs. Panel (i) is reprinted with permission from ref. [80], Copyright 2021, Elsevier. Panel (ii) is reprinted with permission from ref [81], Copyright 2019, American Chemical Society. Panel (iii) is reproduced with permission from ref. [82], Copyright 2020, Elsevier. Panel (iv) is reproduced with permission from ref. [83], Copyright 2023, Wiley‐VCH.

Defects in TMDs have been proven to act as storage/adsorption/active sites for accelerating the electrochemical reactions, whose major effects are summarized in Figure 4.

#### 3.1.1 Promoting Ionic/Electrical Conductivity

Ion diffusion and electron transfer efficiency across the electrode materials largely determine the reversible capacity and electrochemical reaction kinetics of an energy storage device. Defects like vacancies or heteroatom doping generally induce lattice distortion and change the coordination states of nearby atoms, providing additional channels and defect levels for ion diffusion and charge transfer [84]. Various works have evidenced the positive impacts of defects in reaction kinetics. For example, Li et al. developed a self‐supported MoS_2_ nanosheet rich in S vacancies and lattice oxygen [85], which helps enlarge the interlayer distance, and provides additional entrances for ion diffusion and storage, enabling rapid ion insertion into the MoS_2_ lattice not only between the layers but also through the defect. Wen et al. verified that the introduction of Se dopants within the NbS_2_ lattice gives rise to localized electrons and donor levels between the valence band and the conduction band, which narrows the band gap and boosts the concentration of free charge carriers, thereby enhancing the electrical conductivity.^[^ ^86^ ^]^ Furthermore, defects have the potential to lower the energy barrier for the formation of metallic yet thermodynamically unstable phases, such as 1T‐ and 1T’‐MoS_2_, which increase the electric conductivity and introduce additional phase boundaries for ion transport. As demonstrated, the incorporation of 25% 1T‐MoS_2_ phase in 2H‐MoS_2_ nanosheets can greatly increase the electron carrier concentration by an order of magnitude [87].

#### 3.1.2 Increasing the Active Sites

Despite the large specific surface area of few‐layer or monolayer 2D TMDs, a substantial proportion of the surface area is held by the fully‐coordinated basal plane, which is generally electrochemically inert [88]. The most straightforward activation approach is to construct dendritic or porous structures that can expose a high proportion of edges. For example, Gao et al. obtained a vertically oriented ReS_2_ film with sharp exposed edges as polysulfide immobilizers and electrochemical catalysts in the Li–S battery, which significantly improves the conversion reaction kinetics and stability [89]. Another way is to activate the basal plane of TMDs by introducing defects within the in‐plane lattice. A typical case study is chalcogen vacancies in TMDs, which have been widely confirmed to enhance the adsorption capability of cations, anions, and molecules like polysulfides, thereby facilitating the host ion storage, ion dissociation, and conversion reaction. Substitutions like Co, Ni, Cu, and Zn at the metal sites of MoS_2_ have also been verified to possess higher adsorption energies of cations near the doped sites for rapid ion diffusion [90]. However, the defect distribution and concentration should be well considered, as the structural stability may be deteriorated with the increase in lattice distortion. A typical example is the excessive doping phenomena in Mo‐doped VS_2_, where only 5% Mo‐doping concentration exhibits the optimal electrochemical performance [91].

#### 3.1.3 Maintaining Structural Stability

When TMDs are employed as electrode materials in metal‐ion battery electrodes, the reversible intercalation and deintercalation reactions act as the core mechanism for energy storage, which involves a certain degree of volume variation. However, the repeated electrochemical reaction would cause structural degradation and the formation of a thick solid‐electrolyte interphase, leading to the rapid capacity decay [92]. Creating defects within the TMD lattice has been recently explored to achieve structural flexibility and stability of the electrode materials. As mentioned in Section 3.1.1, defects like in‐plane lattice dopants or intercalations can expand the vdW gap for rapid ion diffusion, which also enables the alleviation of volume variation for repeated ion intercalation/deintercalation along the expanded channel, thereby promoting the structural stability of TMDs. Besides, the cation vacancies in TiS_2_ have been found to regulate the sites for alkali metal ion insertion, which greatly reduces the lattice expansion ratio from 37.4% to 11.3% to maintain structural stability [82].

#### 3.1.4 Inducing Spin Effect

Defects can alter the intrinsic spin states of TMDs, which have a significant impact on the bonding, hybridization ability, and charge transport properties of TMDs when employed as electrode materials and catalysts [93]. Typically, TMDs with high lattice symmetry, like MoS_2_, NbS_2,_ and WS_2,_ are inherently non‐magnetic, but the existence of vacancies or foreign atoms can introduce localized states near the Fermi level to accommodate unpaired electrons and induce spin polarization. For example, Li et al. demonstrated that the introduction of Co^2+^ in MoS_2_ introduces negative charges to the layers, which enhances the spin‐polarized surface capacitance and speeds up Li/Na/K‐ion transport across the electrode surface, exhibiting one of the best MoS_2_‐based anodes reported [83]. Defect‐induced spin polarization has also been utilized to alter the adsorption behavior and reaction kinetics between the TMD catalysts and key intermediates in metal–sulfur and metal–air batteries [94, 95, 96].

### 3.2 Applications of Defective TMDs in Electrochemical Energy Storage Devices

**FIGURE 5:**
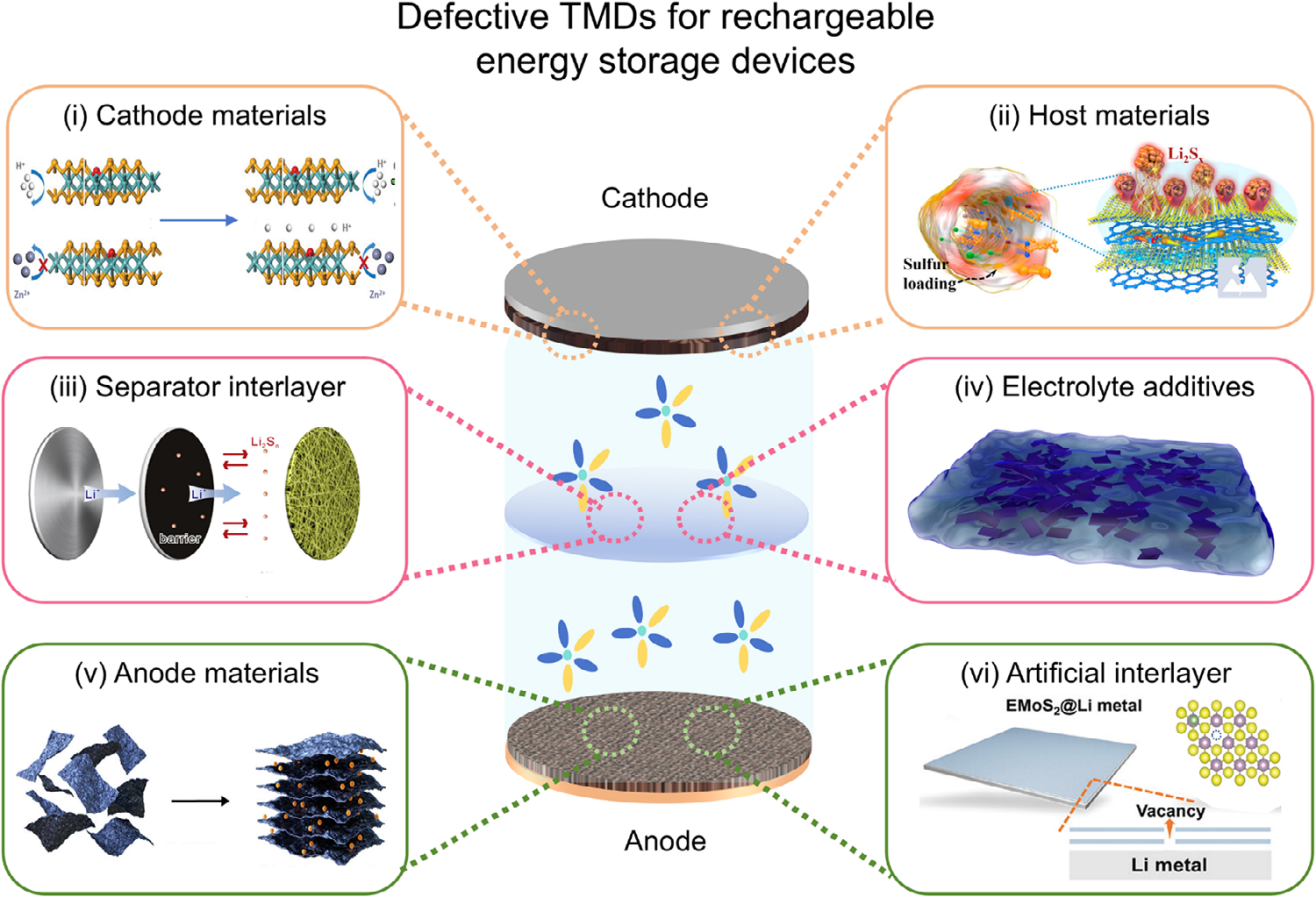
A summary of defective TMDs applied in different parts of rechargeable energy storage devices. Panel (i) is reprinted with permission from ref [97], Copyright 2022, Wiley‐VCH. Panel (ii) is reprinted with permission from ref [98], Copyright 2020, American Chemical Society. Panel (iii) is reprinted with permission from ref [99], Copyright 2018, Cell Press. Panel (iv) is reprinted with permission from ref [100], Copyright 2022, Elsevier. Pannel (v) is reprinted with permission from ref [101], Copyright 2024, Wiley‐VCH. Pannel (vi) is reprinted with permission from ref [102], Copyright 2024, Wiley‐VCH.

Featuring those effects mentioned above, the defective TMDs have been widely employed as electrode materials, bifunctional catalytic electrode materials, artificial interlayers, and electrolyte additives in high‐performance energy storage devices, such as metal‐ion batteries, metal–sulfur batteries, solid‐state batteries, and supercapacitors, etc., which are summarized in Figure 5 and Table 1.

**TABLE 1:** Summary of typical defect engineering on TMDs and their applications in sustainable energy storage devices.

Applications		Substrate/defect types	Synthesis method	Electrochemical performance	Refs.
Electrode materials	Anode materials in LIBs	MoS_2_/Si‐, Fe‐, Cu‐, and O‐ dopants	Natural exist	1 mg·cm^−2^/823 mAh·g^−1^ at 1000 A·g^−1^/72% capacity retention after 1000 cycles	[105]
	Anode materials in SIBs	MoS_2_/Mo‐vacancies	Etching in HCl solution	695 mAh·g^−1^ at 100 mA·g^−1^/88% capacity retention after 100 cycles	[106]
	Anode materials in SIBs	MoS_2_/S‐vacancies and boundaries	Quenching	1.25 mg·cm^−2^/262 mAh·g^−1^at 5000 mA·g^−1^/ nearly 100% capacity retention after 1000 cycles	[107]
	Anode materials in SIBs	MoS_2_/Fe‐dopants	High‐temperature solid‐state reaction	1.2 mg·cm^−2^/680 mAh·g^−1^ at 10 C/84.1% capacity retention after 3000 cycles	[109]
	Anode materials in SIBs	MoSe_2_/Se‐vacancies and P‐dopants	Plasma treatment	328 mAh·g^−1^ at 5000 mA·g^−1^/nearly 100% capacity retention after 1000 cycles	[110]
	Anode materials in KIBs	VSe_2_/Se‐vacancies	Plasma treatment	1.3 mg·cm^−2^/55 mAh·g^−1^ at 3000 mA·g^−1^/92.2% capacity retention after 1000 cycles/206.8 Wh·kg^−1^	[108]
	Anode materials in KIBs	TiS_2_/Ti‐vacancies	Annealing	124 mAh·g^−1^ at 50 mA·g^−1^/63.6% capacity retention after 450 cycles	[82]
	Cathode materials in ZIBs	VSe_2_/Se‐vacancies and O‐dopants	Hydrothermal	2.0 mg·cm^−2^/102 mAh·g^−1^ at 10000 mA·g^−1^/90.5 % capacity retention after 1000 cycles	[85]
	Cathode materials in MIBs	MoS_2_/Li‐intercalation	Chemical reaction	1.0 mg·cm^−2^/221 mAh·g^−1^ at 15 C/ 63.7% capacity retention after 2800 cycles	[113]
	Electrode materials in SC	NiSe_2_/Se‐vacancies and edges	Plasma‐assisted dry exfoliation	466 F·g^−1^ at 3000 mA·g^−1^/81.3% capacity retention after 1000 cycles/19.3 Wh kg^−1^	[121]
	Electrode materials in SC	MoS_2_/Ni‐dopants	Hydrothermal reaction	157 mF·cm^−2^ at 4 mA·cm^−2^/ 77.8% capacity retention after 5000 cycles	[122]
Catalysts	Cathodic catalyst in Li–S batteries	WSe_2_/Se‐vacancies and edge dislocations	Annealing	1.5 mg·cm^−2^ (Sulfur loading)/903.2 mAh·g^−1^ at 1 C/ 82.1% capacity retention after 1000 cycles	[127]
	Cathodic catalyst in Li–S batteries	MoS_2_/Li‐intercalations	Chemical reaction	7.5 mg·cm^−2^ (Sulfur loading)/8 mAh·cm^−2^ at 2 mA·cm^−2^/ 85.2% capacity retention after 200 cycles/441 Wh·kg^−1^	[129]
	Cathodic catalyst in Na–S batteries	MoS_2_/Single Mo‐adatoms	In situ electrochemical reaction	1.0 mg·cm^−2 (^Sulfur loading)/612 mAh·g^−1^ at 1000 mA·g^−1^/72.1% capacity retention after 400 cycles	[130]
	Cathodic catalyst in Li–O_2_ batteries	MoS_2_/In–O‐dopants and Ru nano clusters	Hydrothermal reaction	0.5 mg·cm^−2^/19800 mAh·g^−1^ at 200 mA·g^−1^/284 cycles	[135]
	Cathodic catalyst in Li–O_2_ batteries	MoS_2_/S‐vacancies	Chemical reaction	19 989 mAh·g^−1^ at 200 mA·g^−1^/355 cycles	[134]
Artificial interlayer	Protective layer on Li metal	MoS_2_/Li‐intercalation	In situ diffusion reaction	4.1 mAh·cm^−2^ at 0.1 CC&1 CD/200 cycles/294 Wh·kg^−1^	[141]
	Protective layer on Li metal	MoS_2_/S‐vacancies	Hydrolysis reaction of Li_X_MoS_2_	20 mg·cm^−2^ (NCM cathode)/3.6 mAh·cm^−2^ at 0.5 C/ 91.7% capacity retention after 350 cycles/403 Wh·kg^−1^	[102]
	Interlayer on the current collector	PtSe_2_/Edges	Vapor sulfofication strategy	1.5 mg·cm^−2 (^LFP cathode)/99.1 mAh·g^−1^ at 100 mA·g^−1^/97.8% capacity retention with after 300 cycles	[143]
	Interlayer on the current collector	MoS_X_Se_2−x_/ Anion vacancies	Reduction reaction via hydrazine hydrate	6.0 mg cm^−2^ (sulfur cathode)/813 mAh·g^−1^ at 0.5 C/80.5% capacity retention after 200 cycles/302 Wh·kg^−1^	[142]
Electrolyte additives	Additives in polymer electrolyte	MoSe_2_/edges	In situ selenization route	8 mg·cm^−2^ (NCM cathode)/170 mAh·g^−1^ at 0.5 C/78.5% capacity retention after 110 cycles/	[148]
	Additives in polymer electrolyte	WS_2_/S vacancies	Etching in Ar/H_2_ atmosphere	2 mg·cm^−2^ (Sulfur cathode)/885 mAh·g^−1^ at 0.5 C/60% capacity retention after 500 cycles/435 Wh·kg^−1^	[149]
	Additives in polymer electrolyte	MoS_2_/Li intercalation	Chemical reaction	7.5 mg·cm^−2^ (Sulfur cathode)/1213 mAh·g^−1^ at 0.1 C/85% capacity retention after 200 cycles/350 Wh·kg^−1^	[150]

#### 3.2.1 Electrode Materials

Layered TMDs have been demonstrated to store ions not only through intercalation chemistry, but also via a reversible conversion reaction between M and M_2_X, serving as promising anode materials to promote the energy density of LIBs [103]. Besides, the large interlayer spacing of TMDs theoretically makes it easy to accommodate other alkali metal ions (Na^+^, K^+^), and nontoxic multivalent ions (Zn^2+^, Mg^2+^, Al^3+^), bringing opportunities to build low‐cost metal ion batteries beyond LIBs, such as sodium ion batteries (SIBs) and aqueous zinc ion batteries (ZIBs) [24]. Despite that, great challenges remain for the commercialization of layered TMDs as electrode materials, which can be mainly attributed to their sluggish kinetics, poor structural stability, and low electrical conductivity. Recently, significant progress has been made through defect engineering of TMDs to address these issues. This section primarily introduces the application of defective TMDs as electrode materials in various types of energy storage devices.

**FIGURE 6:**
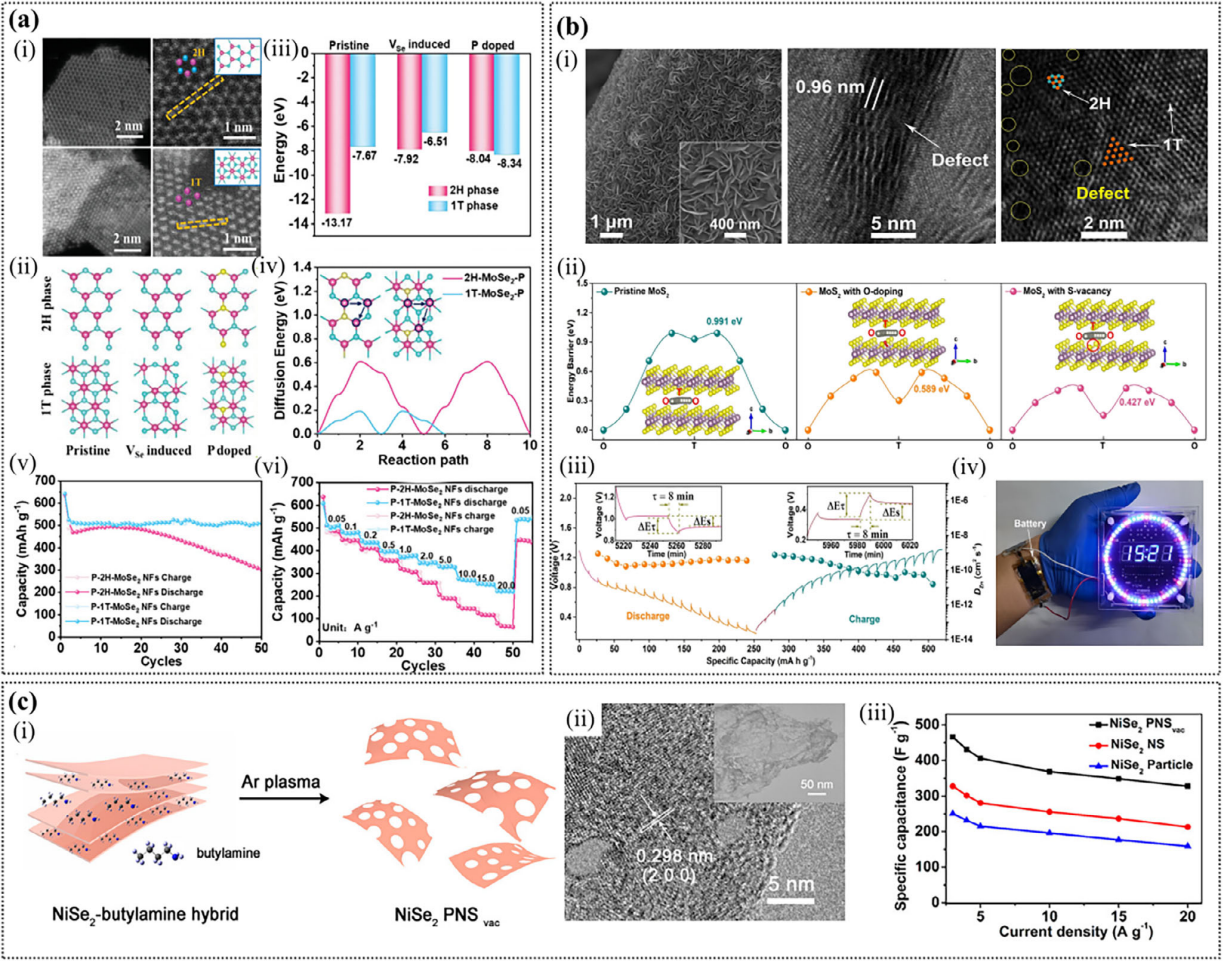
Typical examples of defect engineering on TMDs for their applications as electrode materials in metal‐ion batteries. (a) Atomic structure of defective MoSe_2_ nanosheets rich in vacancies and P dopants, and their theoretical properties and electrochemical performance when employed as anode materials in SIBs [110]. Copyright 2022, Wiley‐VCH. (b) Atomic structure of defective MoS_2_ nanosheets rich in vacancies and O dopants, and their theoretical properties and electrochemical performance when employed as cathode materials in ZIBs [85]. Copyright 2021, Wiley‐VCH. (c) Atomic structure of defective NiSe_2_ nanosheets rich in vacancies and nanopores, and their electrochemical performance when employed as electrode materials in SCs [121]. Copyright 2018, American Chemical Society.

Molybdenite (MoS_2_) naturally exists in the crust with certain impurities [104]. When directly applied as first‐hand electrode materials, molybdenite shows an initial charge capacity of 1199 mAh·g^−1^ and a capacity retention of 72% after 1000 cycles, which is much higher than that of chemosynthetic pure MoS_2_ [105]. Detailed investigation indicates that the natural molybdenite possesses trace amounts of heterogeneous dopants like Si, Fe, Cu, and O, which leads to increased interlayer space and electron density with tuned ionic diffusion kinetics and electron conductivity. However, it is rather difficult to control the concentration and distribution of these impurities within natural minerals. To precisely introduce defects in TMDs, Li et al. utilized a facial selective etching method to create Mo vacancies in MoS_2_ nanosheets, enabling the expansion of the interlayer gap from 0.62 to 0.66 nm to facilitate ion intercalation and buffer the volume changes [106]. Theoretical calculation demonstrates the narrowed bandgap and enhanced reactivity of the Mo vacancies for Na^+^ storage, thereby enabling rapid ion diffusion and improved reversibility. Defective MoS_2_ with abundant vacancies and boundaries has also been successfully synthesized through a facile quenching method [107], showing promoted rate performance and stability compared to that of pristine MoS_2_ at all tested current densities. Beyond intercalation chemistry, the conversion reaction at deeper discharge process generally contributes to more capacity but shows poor reversibility due to the large energy barrier required for bond reconstruction. Sun et al. demonstrated that the introduction of Se vacancies in VSe_2_ nanosheet via an efficient argon plasma treatment not only improves the K^+^ diffusion kinetics by creating new diffusion pathways across the surface, but also induces electron delocalization to weaken the V─Se bond, thereby facilitating the conversion reaction with doubled reversible capacity and lifespan compared to that of pure VSe_2_ [108]. Yu et al. proposed monolayered MoS_2_ with Fe dopants as anode materials via pyrolysis of the precursor containing Fe ions, showing high capacity, excellent fast‐charging performances, and long cycling stability over a wide working temperature. In situ TEM reveals the formation of superparamagnetic Fe nanodomains during the initial discharge process, which triggers strong spin‐polarized surface capacitance that promotes the reversibility of the conversion reaction [109]. In addition, defects potentially lower the energy barrier for initiating the phase transformation from the partial semiconductive phase to the metallic phase, which further optimizes the charge transfer process. Zhang et al. successfully achieved phase engineering in MoSe_2_ nanoflakes through a novel plasma‐assisted P doping approach (Figure 6a(i) and (ii)), where the plasma‐induced Se vacancies not only reduce the energy barrier for the phase transition from the 2H phase to the 1T phase but also facilitate a high concentration of P dopants to function as electron donors for the stabilization of the metastable 1T phase (Figure 6a(iii) and (iv)) [110]. As expected, the P‐doped 1T‐MoSe_2_ shows a high reversibility (a capacity retention of 100% after 1000 cycles, Figure 6a(v)) and rate performance (Figure 6a(vi)) for Na^+^ storage.

Owing to the large interlayer space with weak interactions, layered TMDs have also been verified as ideal host materials to intercalate multivalent ions in aqueous electrolytes with a moderate working potential when paired with Zn, Mg, or Al metals as the anode, showing incomparable advantages of low cost, safety, and environmental friendliness when compared to non‐aqueous battery systems [111]. For example, Li et al. unlocked the basal plane of MoS_2_ via creating S vacancies and O dopants (Figure 6b(i)) during the hydrothermal synthesis process [85], exhibiting high reversible capacities of 261 and 102.4 mAh·g^−1^ at 0.1 and 10 A·g^−1^, respectively, and remarkable cycle stability with 90.5% capacity retention after 1000 cycles when employed as cathode materials in wearable Zn batteries (Figure 6b(iv)). The enhanced electrochemical performance can be attributed to the synergistic effects of vacancies and O dopants that reduce the ion diffusion barrier (Figure 6b(ii) and (iii)), enlarge the interlayer space, improve the hydrophilicity, and stabilize the metallic 1T phase with high electronic conductivity. Self‐supported VSe_2_ nanosheets rich in Se vacancies were also reported for Zn^2+^ storage [112], showing a satisfactory reversible capacity of 265.2 mAh·g^−1^ at 0.2 A·g^−1^ with impressive cyclic stability. Theoretical calculation revealed that the presence of Se vacancies modulates the adsorption energy of Zn^2+^, thereby making the electrochemical reaction more reversible. Similar defect engineering strategies and modulation effects in TMDs can be extended to other aqueous battery systems, including Mg‐ and Al‐ion batteries [113, 114].

The extremely large surface area and tunable vdWs gap of TMDs also facilitate the Faradaic charge transfer process, which breaks the limitations of diffusion‐controlled processes in battery systems to construct supercapacitors featuring both high energy density and power density [115, 116, 117]. The most prevalent strategy for optimizing Faradaic materials is to maximize the exposure proportion of their edges. Various nanostructured transition metal dichalcogenides (TMDs), such as quantum dots, nanosheets, nanocoral, and nanowires, have been developed in recent years [78, 118, 119]. For example, Gong et al. developed a self‐supported electrode based on Ni_0.85_Se@MoSe_2_ nanosheet arrays via a facile one‐step hydrothermal method for asymmetric supercapacitors, showing a specific energy of 19.3 Wh·kg^−1^ at an ultrahigh power of 3900 W·kg^−1^ [120]. To further activate the basal plane, Chang et al. employed Ar plasma treatment on lamellar NiSe_2_‐butylamine hybrid precursors (Figure 6c(i)) to directly exfoliate NiSe_2_ and induce pores and Se vacancies (Figure 6c(ii)), showing a significantly promoted capacitance (466 F·g^−1^) and cycling stability in 1 M KOH electrolyte (Figure 6c(iii)) compared to that of NiSe_2_ particles or perfect NiSe_2_ nanosheets [121]. Panghal et al. optimized the capacitance of the MoS_2_ electrode by introducing different Ni doping concentrations during the hydrothermal reaction process. Specifically, the 10% Ni‐doped MoS_2_ exhibits a capacitance 1.5 times greater than that of pure MoS_2_, which can be attributed to the balance in active sites and structural stability with moderate Ni concentration [122].

#### 3.2.2 Bifunctional Catalytic Electrode Materials

Conversion‐type metal batteries, such as Li–S and Li–air batteries, exhibit theoretical energy densities that are over 10 times higher than those of current state‐of‐the‐art Li‐ion batteries, which have been extensively regarded as one of the most promising candidates for next‐generation battery systems [123, 124]. However, the practicability of the conversion‐type cathodes has been hindered by a myriad of crucial challenges, including the insulating nature, volume change, slow reaction kinetics, and shuttling effect. Typically, the redox process of these batteries involves multiple conversion steps at the cathode side, all of which require conquering a certain energy barrier [125]. Progress has been made on designing efficient cathode catalysts to promote the reversibility and kinetics of the redox reaction, particularly on 2D TMDs with tunable surface activity and electronic structure. For pristine TMDs, the catalytic centers are concentrated around the edge sites, which inspires the design of a dendritic structure or hierarchical porous structure to maximize the active sites. In addition, diverse defect configurations have also been introduced to further activate the basal plane of TMDs for efficient redox reaction. In this part, the current advances regarding defective TMDs in different conversion‐type metal batteries are elaborated.

**FIGURE 7:**
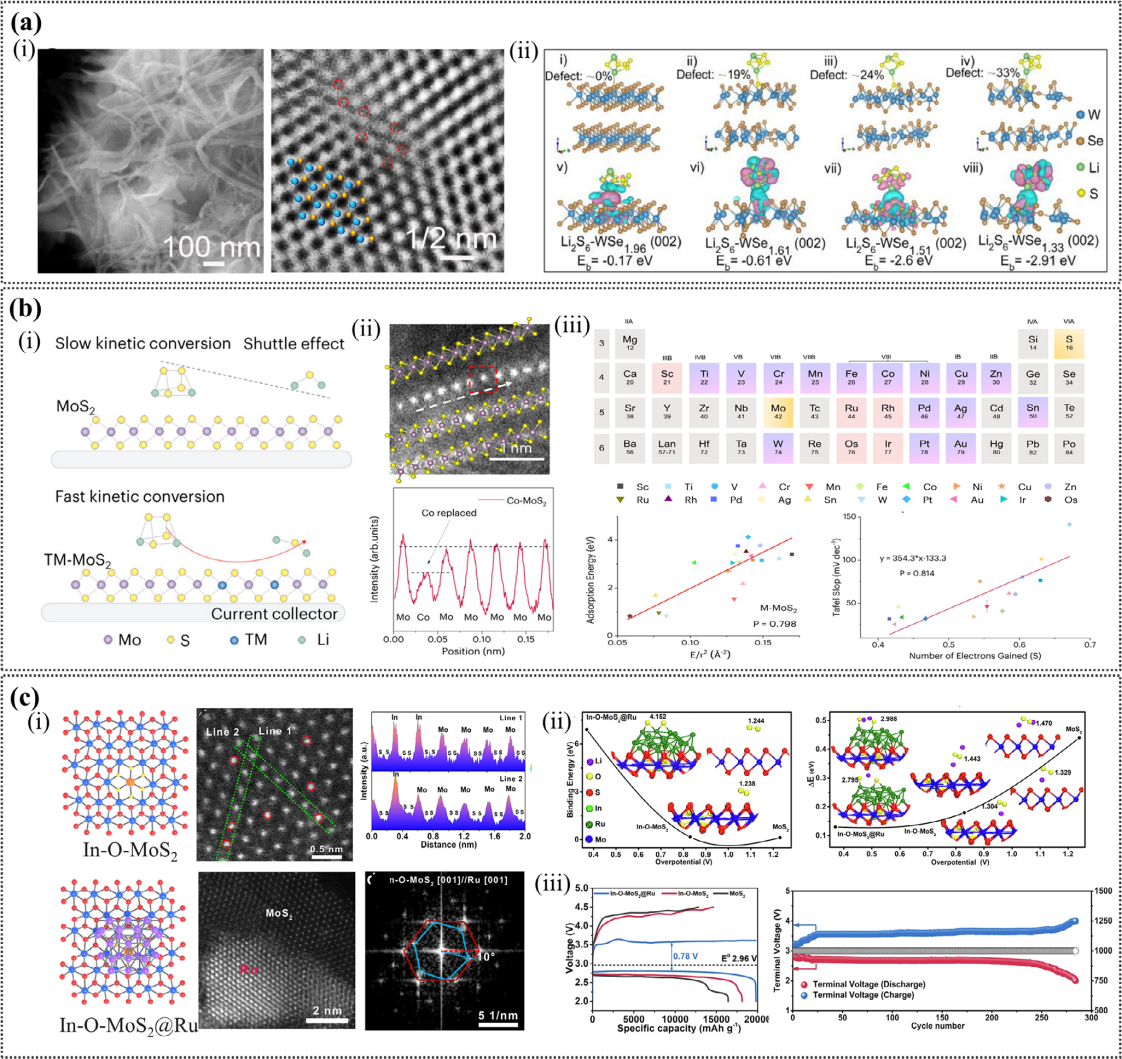
Typical examples of defect engineering on TMDs for their applications as catalysts in conversion‐type metal batteries. (a) Atomic structure of defective WSe_2−x_ nanosheets rich in anionic Se vacancies and edge dislocations, and their theoretically catalytic properties when employed as catalytic host materials in Li–S batteries [127]. Copyright 2022, Elsevier. (b) Atomic structure of MoS_2_ nanosheets with transition metal dopants and their relationships between lattice sulfur electron density and sulfur species reduction activity when employed as catalytic host materials in Li–S batteries [128]. Copyright 2025, American Chemical Society. (c) Atomic structure of MoS_2_ nanosheets rich in In–O dopants and Ru nanocrystalline, and their theoretically catalytic properties and electrochemical performance when employed as cathode catalyst materials in Li–air batteries [135]. Copyright 2025, Springer Nature.

Elemental chalcogens, such as sulfur and selenium, have been confirmed as high‐energy‐density cathodes in Li, Na, and Zn metal batteries, which share similarities in the energy storage mechanisms, yet encounter a primary challenge of severe shuttling effect. The shuttling effect originates from the formation of dissolvable intermediate species that gradually diffuse to the anode side via the electrolyte, leading to severe self‐discharge phenomena and rapid capacity decay [126]. The most straightforward approach is to build blocks on the separators or design efficient hosts with defective TMDs to enhance the adsorption capability of the intermediate species. For example, A series of defective WSe_2−x_ nanosheets rich in anionic Se vacancies and edge dislocations (Figure 7a(i)) was employed as efficient catalysts in 3D conductive hosts for high‐performance Li–S batteries [127]. Theoretical calculations verify the enhanced adsorption energy of the polysulfide with the increase in defect concentration (Figure 7a(ii)), which can be attributed to the atomic rearrangements around the defect sites that provide additional S_x_^2−^‐W^δ+^ coupling interaction. However, excessive bonding strength with the polysulfide will deteriorate the conversion kinetics and, in turn, decrease the utilization of active sulfur during the redox reaction process. To search for a balance between the adsorption capability and redox kinetics (Figure 7b(i)), Zhang et al. provide a theoretical guideline for defect design in MoS_2_ by exploring the relationships between lattice sulfur electron density and sulfur species reduction activity within a series of transition metal‐doped MoS_2_ catalyst systems [128]. As demonstrated, the substitution of Mo atoms with other heteroatoms disrupts the electronic symmetry of MoS_2_, which promotes the transfer of spin‐polarized electrons from metal centers to lattice sulfur. More interestingly, it was found that the electron transfer number exhibits a direct linear relationship with both the Tafel slope and the adsorption energy in sulfur conversion chemistry (Figure 7b(iii)). Therefore, transition metals like Co (Figure 7b(ii)), Cr, and Pt that simultaneously exhibit higher adsorption energy and a smaller Tafel slope can be precisely selected as promising candidates for the construction of high‐efficiency catalysts in Li–S batteries. Given the electronic insulating feature of polysulfide species, a pre‐lithiated metallic 1T‐MoS_2_ was directly employed as a binder‐free host material for high‐performance Li–S batteries under lean electrolyte conditions [129]. As experimentally verified, the intercalated lithium in Li_x_MoS_2_ creates abundant binding sites to facilitate the adsorption of polysulfides. It also offers a conductive pathway to enhance Li^+^ transport and serves as a lithium reservoir, thereby enabling more efficient electrochemical reaction kinetics. Assisted by the Li_x_MoS_2_ host, an Ah‐level Li–S pouch cell was successfully constructed, featuring both high gravimetric energy density of 441 Wh·kg^−1^ and capacity retention of 85% even after 200 cycles. Furthermore, it was found that a deeper sodiation process of MoS_2_ can trigger the formation of elemental Mo distributed in a single‐atom form on the substrate, which can serve as catalytic centers for accelerating sulfur redox chemistry of room‐temperature Na–S batteries [130].

Metal–air batteries (MABs), especially Li–O_2_, Na–O_2_, and Zn–O_2_ batteries, possess the potential to extend the energy density limits of battery systems to a level comparable to that of fuels, which holds great promise for meeting the increased energy demands. In a typical Li–O_2_ battery system, the overall electrochemical process involves the oxygen reduction reaction (ORR) during discharge and oxygen evolution reaction (OER) during charge, delivering a theoretical energy density of 3500 Wh kg^−1^. Unfortunately, the sluggish redox kinetics and inferior reversibility of superoxides significantly deteriorate the round‐trip efficiency and cycling stability of MABs, which motivates researchers to develop an efficient cathode catalyst for accelerating the redox reaction process [131]. Recently, 2D TMDs like MoS_2_ and MoSe_2_ have demonstrated high catalytic activity in the direct formation and decomposition of Li_2_O_2_ when employed as bifunctional catalytic electrode materials in Li–O_2_ batteries [132, 133]. Diversified defect configurations have also been extensively introduced to enrich the active sites of TMDs. For example, vacancies were created in MoS_2_ nanosheets via a NaBH_4_ reduction post‐processing, which shifts the electronic structure of the substrate from semiconductor to conductor [134], while strengthening the interaction with the intermediates on the cathode, leading to the formation of an ultrathin Li_2−x_O_2_ film with better electrical conductivity and reversibility. When incorporated into 3D conductive frameworks, the composite catalyst enables Li–O_2_ battery cycling at 1000 mA g^−1^ with a specific capacity of 500 mAh·g^−1^ for more than 600 cycles. To further enhance the electronic conductivity, p‐block element (In–O) doping strategy was employed to stabilize metallic T‐phase MoS_2_, which simultaneously strengthens the internal chemical bonding for the epitaxial Ru nanocatalyst graft on the stabilized substrate (Figure 7c(i)) [135]. The composite catalyst with robust lattice‐grafting hetero‐interface greatly facilitates the formation of amorphous Li_2_O_2_ films during ORR, thereby efficiently decreasing the redox barriers (Figure 7c(ii)) and enhancing the bifunctional catalytic stability (Figure 7c(iii)). Besides, Li–CO_2_ batteries can be regarded as a specific subtype of MAB in a broad sense, which involves a CO_2_ reduction (CO_2_RR) and CO_2_ evolution reactions (CO_2_ER) similar to those of Li–O_2_ batteries. To efficiently catalyze the redox reaction, ReS_2_ nanosheets rich in nucleophilic N dopants and electrophilic S vacancies were created via hydrothermal reaction to simultaneously tailor the interactions with Li atoms and the C/O atoms in intermediates [136]. As theoretically demonstrated, the electrophilic and nucleophilic dual centers exhibit appropriate adsorption with all intermediates during both the discharge and charge processes, contributing to a relatively small energy barrier for the rate‐determining step. Assisted by the optimal catalyst with dual active centers, the Li–CO_2_ battery achieves an ultrasmall voltage gap of 0.66 V and ultrahigh energy efficiency of 81.1% at 20 µA·cm^−2^ for catalyzing CO_2_ redox reaction.

#### 3.2.3 Artificial Interlayer

**FIGURE 8:**
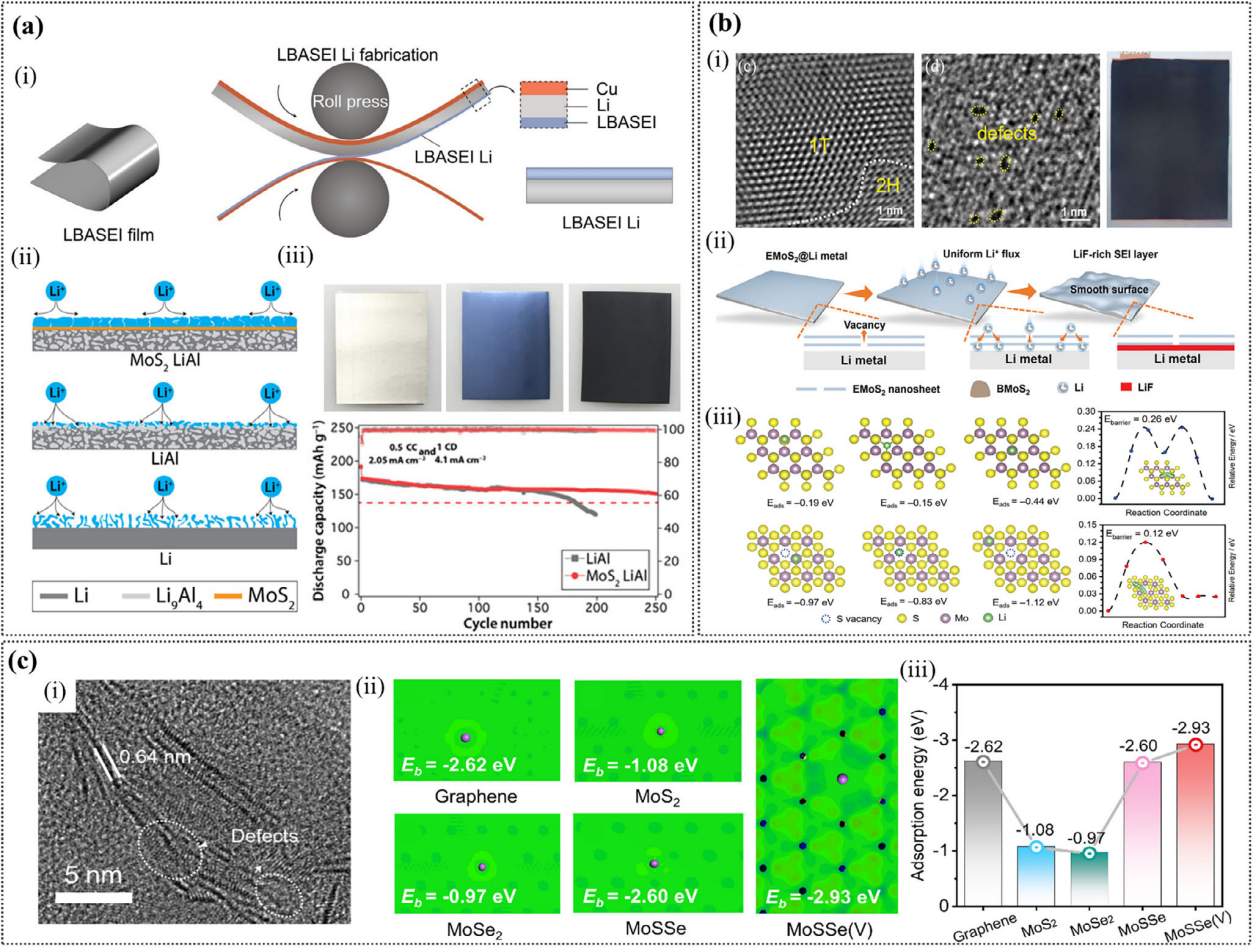
Typical examples of defect engineering on TMDs for their applications as an artificial interlayer on metal anodes. (a) Schematics showing the transfer process of Li‐intercalated 1T‐MoS_2_ film on Li metal anode, its modulation effects during the Li deposition process, and the electrochemical performance of the full cells with protected Li metal anode [141]. Copyright 2019, American Association for the Advancement of Science. (b) Atomic structure of vacancy‐rich 1T‐MoS_2_, its modulation effects during the Li deposition process, and the Li^+^ diffusion barrier across different interlayers [102]. Copyright 2024, Wiley‐VCH. (c) Atomic structure of MoS_x_Se_2−x_ alloys rich in anionic vacancies and the binding energy of Li+ with different adsorption sites [142]. Copyright 2022, American Chemical Society.

Metal‐based batteries, such as those with metallic Li, Na, Zn, etc. as anode, possess much higher energy density compared to their respective ion‐based battery systems [137, 138, 139, 140]. However, uncontrollable dendrite formation as well as severe side reactions are inevitable during the repeated plating/stripping process, leading to the rapid capacity decay and safety concerns. Essentially, dendrite formation originated from the uneven ion distribution at the electrolyte/electrode interface, which can be significantly alleviated by introducing a robust artificial interlayer on the metal electrode surface. Considerable efforts have recently been devoted to employing 2D TMDs as an artificial interlayer for stabilizing metal anodes, particularly when defects are elaborately involved. For example, a layer‐by‐layer assembled 1T‐phase MoS_2_ film with Li intercalation dopants [141] was designed on a Li‐based anode via the Langmuir–Blodgett method (Figure 8a(i)), followed by a mechanical transfer process. Both theoretical calculation and electrochemical experiments demonstrate the significantly reduced diffusion barrier for Li ions across the 1T‐MoS_2_ layer (Figure 8a(ii)), enabling uniform Li deposition beneath the protective layer with nearly two‐fold extension of the lifespan in both symmetric and full cells (Figure 8a(iii)). Similarly, Choi et al. established a layer of Li‐intercalated MoS_2_ via sputtering [135], which induces a phase transformation of MoS_2_ from the 2H to the 1T phase, promoting electric conductivity and thereby reducing the local current distribution with uniform Li deposition, even at a high current density of 10 mA cm^−2^. To further activate the basal plane, Huang et al. designed a defective MoS_2_ nanosheet rich in S vacancies and 1T phase (Figure 8b(i)) as a protective layer for stabilizing the Li metal anode (Figure 8b(ii)) [102]. Theoretical calculation demonstrated the positive role of vacancies in trapping anions while increasing the affinity of Li^+^, thereby reducing the ion gradient near the anode with homogeneous ion flux during lithium plating (Figure 8b(iii)). Furthermore, the ultrathin MoS_2_ layer exhibits an average Young's modulus of 17.8 GPa, which is three times higher than the threshold to suppress Li dendrite. Protected by this defective interlayer, the Li metal anode shows a long‐term stability of 2000 h even at a high deposition capacity of 4 mAh·cm^−2^ in symmetric cells, laying a good foundation for the construction of 403 Wh·kg^−1^ Li metal batteries with satisfactory cycling stability.

In addition, defective TMDs are capable of reducing the nucleation barrier for the epitaxial growth of metal anodes on a heterogeneous current collector. For example, Wang et al. modified the surface of carbon networks with a layer of vertically‐aligned PtSe_2_ nanoflakes to optimize the Li nucleation behavior [143]. As demonstrated, the Li nucleation barrier can be significantly reduced from 59 to 11 mV at a current density of 5 mA·cm^−2^, which is ascribed to the exposure of active sites along the edges for Li^+^ desolvation, as well as the formation of Li_2_Pt and Li_2_Se nanocrystallines during the prelithiation process that reduce the interfacial energy between Li nuclei and the carbon substrate. As a result, Li metal can undergo uniform nucleation and growth along the 3D conductive networks, extending the lifespan of LiFePO_4_‐based Li metal batteries up to 300 cycles. Anion vacancies were also introduced in MoS_x_Se_2−x_ alloys (Figure 8c(i)) to enhance the interaction between Li^+^ and the substrate, which enhances the affinity of Li^+^ with the substrate (Figure 8c(ii)), thereby facilitating uniform Li nucleation and growth inside the entire rGO frameworks with a long‐term stability of 1200 h in symmetric cells [142].

#### 3.2.4 Electrolyte Additives

**FIGURE 9:**
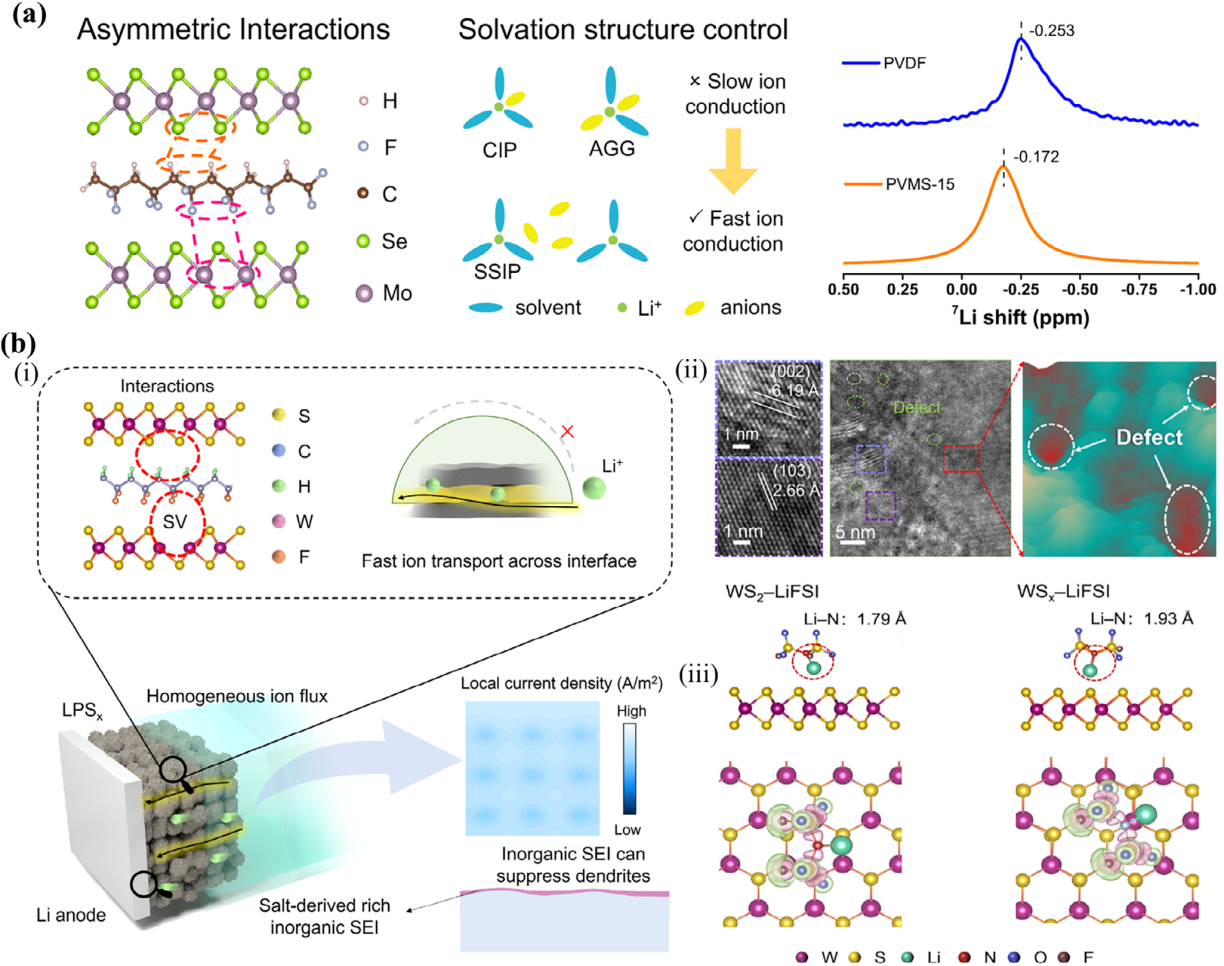
Typical examples of defective TMDs for their applications as an artificial interlayer on metal anodes. (a) Atomic structure showing the interaction between MoSe_2_ and PVDF chains, and the effects of MoSe_2_ nanosheets on the solvation structure modulation [148]. Copyright 2023, Springer Nature. (b) Atomic structure of WS_2_ nanosheets rich in sulfur vacancies, the adsorption energy of FSI^−^ anions at the vacancy sites, and the schematics showing the effects of the defective WS_2_ as fillers in PVDF‐based polymer electrolyte [149]. Copyright 2025, Wiley‐VCH.

As a promising alternative to liquid electrolytes, polymer electrolytes exhibit excellent flexibility and good compatibility with the electrodes, which are expected to match the battery manufacturing processes for mass production [144, 145]. Typically, an ideal polymer electrolyte should possess comparable ionic conductivity and electrochemical window to that of a liquid electrolyte [146]. Nevertheless, challenges like limited ion transport and the unstable solid electrolyte interphase remain when solely employing a polymer matrix, leading to severe polarization and rapid capacity decay of Li metal batteries. Great progress has been delivered to optimize the electrochemical performance of polymer electrolyte via introducing 2D TMDs as fillers into the matrix, which can trigger strong interfacial interaction with the chain segments and Li salt to effectively dissociate Li salt and provide additional ionic channels [147]. For example, 2D MoSe_2_ nanosheets were recently proposed as an efficient additive for the construction of a dense and robust PVDF‐based polymer electrolyte [148]. Interestingly, the positively charged Mo atom has been shown to interact with the ─CF_2_─, whereas the negatively charged Se atoms interact with the ─CH_2_─, facilitating the symmetry disruption of the PVDF matrix with the formation of polar β‐phase (Figure 9a). Therefore, Li salt can be effectively dissociated within the β‐phase PVDF substrate, which enhances the ionic conductivity of the polymer electrolyte and shows robust cycling stability under practical conditions in pouch cells.

To optimize the interfacial interaction, vacancy‐rich WS_2_ nanosheets (Figure 9b(ii)), obtained by an atmosphere etching method, were employed as functional fillers to boost the ionic transport ability of PVDF‐based polymer electrolyte (Figure 9b(i)) [149]. As demonstrated, the in‐plane vacancies not only exhibit a robust electrostatic interaction with the polymer chains to create abundant ion transport interfaces within the electrolyte, but also strongly interact with the FSI^−^ anions (Figure 9b(iii)), thus liberating Li ions and promoting the ionic conductivity of the composite electrolyte up to 1.9 × 10^−3^ S·cm^−1^ at 25°C. Furthermore, the trapped anions tend to preferentially degrade around the vacancy sites, enabling the formation of a robust SEI layer rich in inorganic components. Under these synergistic effects, solid‐state Li metal batteries exhibit remarkable reversibility during the Li plating/stripping process, showing a long‐term stability of 5500 h at 0.1 mA·cm^−2^. However, vacancies in TMDs are not always favorable for upgrading the electrochemical performance of polymer electrolytes. A typical example is the over‐polymerization phenomenon of 1,3‐dioxolane (1,3‐DOL) monomer driven by the increased Lewis acidity of Mo centers in MoS_2_ when surrounded by vacancies, which decreases the unpolymerized liquid content with deteriorated wettability between the electrode and polymer electrolyte. To alleviate this problem, additional electrons were introduced through Li^+^ intercalation to neutralize the Lewis acidity of Mo centers, enabling the retention of 13% unpolymerized liquid 1,3‐DOL in QSSE to maintain the interfacial compatibility and ionic conductivity [150]. Therefore, Li metal anode can be reversibly plated/stripped at a current density of 1 mAh·cm^−2^ over 200 h, which further boosts the construction of high‐energy–density Li–S pouch cells with a three‐fold increase in cycle life over a wide working temperature.

In short, introducing defects into TMDs can enhance their electronic conductivity, provide sufficient binding sites, create new ion‐transfer pathways, and mitigate reaction barriers, thereby improving the electrochemical performance of TMDs in terms of capacity, operating potential, cycling stability, and rate performance for various energy storage applications. However, given the structural diversity of defects in TMDs, not all defect types are beneficial, and their effect on electrochemical performance does not always present a linear relationship with concentration [151]. How to rationally design defects (including defect types, concentrations, and distributions) and maximize their beneficial effects requires an in‐depth investigation by combining computational and experimental studies. Also, the real working states of electrode materials and their effects on defects during the redox reaction remain unclear. For example, in some cases, the defects may disappear after the first cycle and cannot be regenerated, yet the electrochemical performance improvements can still be maintained in the subsequent cycles [152]. Therefore, the exact role of defects in modulating electrochemical performance and their dynamic behavior is worthy of further study using advanced in situ techniques [153, 154].

## 4 Machine Learning (ML) for Intelligent TEM Analytics

Given the diversified atomic structures and modulation effects of defects in TMDs mentioned above, it is imperative to uncover the structure–property correlations for precise defect manipulations, which nowadays can be realized by advanced TEM techniques [155]. Over the past decades, the prosperity of the TEM techniques has produced a large quantity of microscopic data from various types of defects across different material systems, while simultaneously posing new challenges from the following two aspects: (1) limited signal‐to‐noise ratio (SNR) and spatial resolution, especially in the low electron dose scenarios involving the characterization of electron‐sensitive TMDs and transient data collection; (2) immense labor and time required for manual analysis from massive data, as well as the associated bias judgements [156]; Fortunately, the recent advances in ML have been revolutionizing the paradigm of microscopic analytics, providing a powerful tool for high‐throughput TEM investigations. This part gives a comprehensive summary of the cutting‐edge advancements in the field of ML‐driven atomic microscopy data analytics for constructing high‐quality datasets, atomic structure analysis, and new knowledge inspiration.

### 4.1 ML for Constructing High‐Quality Datasets

**FIGURE 10:**
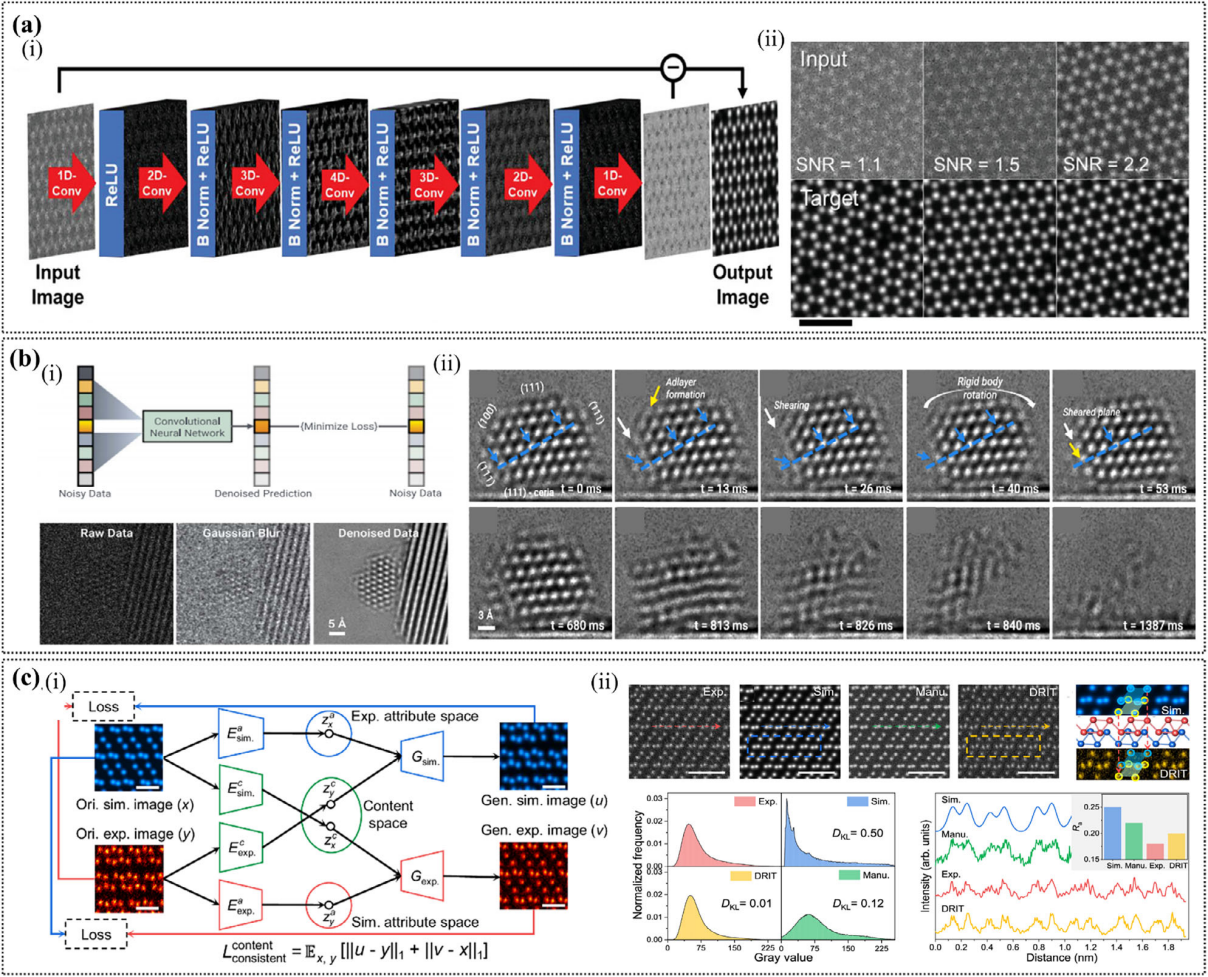
Typical examples of ML for constructing high‐quality datasets. (a) Deep learning‐based denoiser framework and its restored ADF‐STEM images of V‐doped WSe_2_ after the denoising process. Scale bars: 0.5 nm [160]. Copyright 2021, Wiley‐VCH. (b) Unsupervised deep denoising frameworks and the frame‐by‐frame HR‐TEM images of Pt nanoparticle after denoising to describe the formation of a subsurface stacking fault [161]. Copyright 2025, AAAS. (c) Disentangled representation (DR) learning framework and its performance in generating ADF‐STEM images of bilayer ReS_2_. Scale bars: 1 nm [164]. Copyright 2025, Springer Nature.

High‐quality datasets lay the foundation for both manual data analysis and ML models to learn image features. However, noise and aberration are inevitable during the imaging process, which greatly decreases the TEM's resolution ability [157, 158, 159]. The most straightforward way is to develop denoising models to make the raw data clear. For example, Kim et al. built an image restoration algorithm based on convolutional neural networks (CNN), a neural network that utilizes convolutional kernels to extract image features, to reduce the statistical noise and enhance the SNR of STEM images with the aim of dataset preparation for the downstream tasks (Figure 10a(i)) [160]. Simulations with statistical noise signals that follows Poisson distribution were intentionally generated as training datasets to approximate the experimental ADF‐STEM images, and after training, the denoiser algorithm successfully enhances the SNR of the experimental data from 1.1 to 16.1 without the loss of key information (Figure 10a(ii)), which further boots the subsequent defect segmentation algorithm based on fully convolutional network (FCN) with a high measurement accuracy≈98% for the detection of atomic columns and defect sites. Besides, ML models can be trained to function as an “aberration corrector” to improve the spatial resolution of an uncorrected STEM. A diffusion model named SARDiffuse was recently established to alleviate the aberration of low‐resolution STEM images [158]. During the model training process, Gaussian noise was forward added with random steps to the aberration corrected‐STEM (AC‐STEM) images, followed by a backward diffusion process to restore the original images from the noisy ones by utilizing the prior knowledge of the noise‐addition process. Iterative inference from the trained diffusion model enables the restoration of uncorrected STEM images across diversified material systems from the raw resolution of ∼136 pm to better than 100 pm. However, the above‐mentioned models require certain low‐noise and high‐noise data pairs from the same region, which may encounter difficulties concerning high data acquisition cost and limited generality for the “unseen” data. Recently, Crozier et al. developed an unsupervised denoising model based on the U‐Net architecture to track the edge evolution of metal nanoparticles in a gas environment with temporal resolutions as low as 10 milliseconds under moderate electron doses (Figure 10b(i)) [161]. The fundamental principle of this model is grounded in the assumption that noise is independent across frames, where the model estimates each noisy pixel value using the surrounding spatiotemporal neighborhood but without considering the noisy pixel itself, enabling the extraction of the underlying clean image structure without overfitting the noise. With this unsupervised deep denoising framework, the SNR of the outputs has been improved by a factor of ∼36 compared with the raw data, enabling the discovery of transitions between ordered and disordered configurations, as well as the fluidity of nanoparticles induced by defects and stress at a high spatial and temporal resolution (Figure 10b(ii)).

Another approach lies in generating noise similar to that of experimental data on the noise‐free simulation images, which enables a direct alignment of the labels from known structures with the realistic noisy data. For example, Clark et al. employed a generation model named the cycle generative adversarial network (CycleGAN) to produce high‐quality simulation data that is nearly indistinguishable from real data [162]. The CycleGAN model contains a “generator” for high‐quality “fake” image generation from another domain, as well as a “discriminator” to determine whether the output from the generator is real or fake. Repeatably converting images from one domain to another enables the model to augment simulations with realistic spatial frequency information. Therefore, the CycleGAN‐generated simulation image shows the lowest Kullback‐Leibler (KL) divergence of 0.01, which is even below the manually optimized one. This framework has also been extended to generate simulated motifs similar to the experimental images of a defective MoS_2_ rich in vacancies and O‐dopants, by only using one experimental STEM image as the training set, which provides a high‐quality training set upon switching for defect identification tasks [163]. However, the CycleGAN algorithm lacks a true understanding between the content information (referring to the structural information) and the style information (referring to the noise information) within an image, which may lead to structural distortions after style transfer, especially for images containing complex motifs. To further optimize the consistency of image contents, Huang et al. have developed a disentangled representation (DR) learning approach (Figure 10c(i)), which decouples simulated and experimental images into two distinct spaces: a domain‐invariant content space to capture shared structural information, and a domain‐specific attribute space to independently extract the visual styles [164]. Moreover, a trident strategy involving data selection, data augmentation, and the design of a content‐consistent loss function was implemented together on the DRIT model to enhance the structural consistency between the original experimental/simulation image and the generated simulation/experimental images after visual style conversion. By employing a few unlabelled experimental images with abundant low‐cost simulated images, a large corpus of annotated simulation data that closely resembles experimental results in both styles and contents have been successfully generated (Figure 10c(ii)), with which a robust structural inference model is therefore achievable to automatically analyze the interlayer slip and rotation modes from diversified and complicated stacking patterns with picometer‐scale accuracy across various materials (e.g. MoS_2_, WS_2_, ReS_2_, ReSe_2_, and 1 T’‐MoTe_2_), and different layer numbers (bilayer and trilayers). The generative models mentioned above hold great potential for high‐throughput generation of simulation datasets that cover most of the experimental variations, therefore alleviating the dilemma of limited TEM datasets and the time‐consuming manual label process for the downstream ML model training.

### 4.2 ML for Atomic Defect Analysis

**FIGURE 11:**
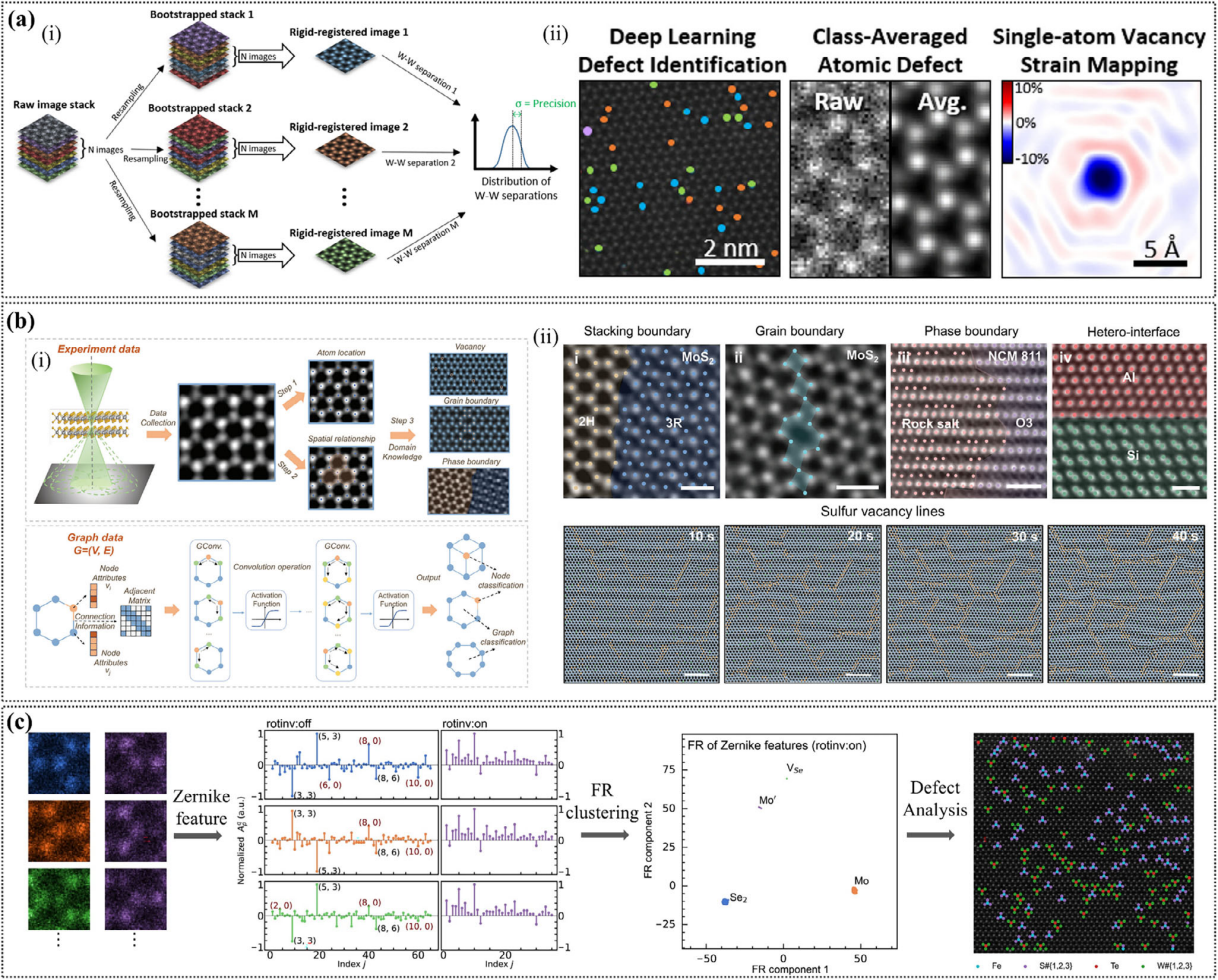
Typical examples of ML for defect identification in atomic resolution microscopy. (a) Schematic illustration of the bootstrapping process for precision measurement of local strain around vacancy sites, and the generated strain map [167]. Copyright 2020, American Chemical Society. (b) Schematic showing the similarity between the manual analysis process of an ADF‐STEM image and the GNN modeling process, and the identification results across a library of defective materials from GNN frameworks [171]. Copyright 2025, Wiley‐VCH. (c) Schematic showing the defect classification process via an unsupervised clustering method using Zernike coefficients as image patch features [173]. Copyright 2022, Wiley‐VCH. AAAS.

Currently, a series of ML models have emerged for high‐throughput TEM data analysis involving atomic pinpointing, atomic column identification, and defect analysis, which have been summarized in Table 2. A pioneering ML model named AtomSegNet, which consists of a typical U‐net architecture and an Otzu algorithm, was established by Xin et al. to localize atomic columns [165]. After being trained with the pre‐established TEM ImageNet library that contains thousands of simulations with varied structures, orientations, as well as noise, the AtomSegNet demonstrates a self‐adaptability to the experimental STEM images and exhibits an outstanding robustness in terms of atom segmentation/localization and edge/surface atom detection. Beyond atomic position recognition, atomic column type and point defects like vacancies and heteroatom dopants can also be automatically processed for statistical analysis. For example, Ziatdinov et al. developed a FCN‐based architecture coupled with a Gaussian blob detection technique [166] to locate and identify multiple defect structures according to the position of chemical species, bond coordination, length, and angle in atomically resolved STEM images. With this elaborately designed framework, only a limited number of available labels are required, and it even enables the identification of the “unseen” defects that are not included in the initial training set. Similarly, Lee et al. trained a DL model based on ResUNet to pinpoint and classify the multiple point defects, including Te substitutions and Se vacancies in WSe_2−2x_Te_2x_ alloy (Figure 11a(i)) [167]. Benefited from the high‐throughput processing capability, class‐averaged reference images of each defect were generated from numerous identified images to measure the 2D displacement vectors, which help map out the 2D strain fields of each single‐atom defect with sub‐picometer precision (Figure 11a(ii)).

**TABLE 2:** Summary of typical ML methodologies on high‐throughput TEM data analytics for atomic defect characterizations in TMDs.

Models	Materials/structures	Defects categories	Refs.
FCN	WSe_2_ monolayer	Vacancies	[162]
FCN	MoS_2_ monolayer and bilayer with different phases	Phase boundaries and vacancies	[168]
CNN	V‐doped WS_2_ monolayer	Dopants and vacancies	[174]
U‐Net	V‐doped WSe_2_ monolayer	Vacancies	[160]
One‐class support vector	W‐doped MoTe_2_ monolayer	Vacancies	[30]
ZP and FR‐assisted clustering	Te‐ and Fe‐doped WS_2_ monolayer, MoSe_2_ bilayer with a continuous stacking pattern	Dopants, vacancies, phase boundaries, grain boundaries, stacking faults	[173]
FCN	WSe_2−x_Te_2x_ alloy	Vacancies	[167]
U‐Net	MoS_2_ monolayer	Vacancies and vacancy lines	[190]
FCN	MoS_2_, WSe_2_, NbSe_2_, and NbS_2_ monolayer	Vacancies	[163]
EGNN	MoS_2_ monolayer and bilayer with different phases	Vacancies, dopants, grain boundaries, phase boundaries, vacancy lines, composite defects	[171]
ZP and UMAP‐assisted clustering	MoS_x_Te_2−x_ alloy	Antisite‐defects	[172]

Beyond the identification of simple point defects, Kim et al. trained a ResUNet‐architected model for reliable identification of stacking configuration from high‐resolution STEM images of layered MoS_2_, which show robustness against carbon contamination and random noise [168]. Given the excessive pixel information beside the atomic columns (like the vacuum region), Zhu et al. trained a model with a hard attention mechanism in conventional U‐Net architectures to guide the network toward the relevant area of interest [169], which outperformed the vanilla models for atom‐by‐atom phase segmentation within interlaced phase domains of a layered cathode material in LIBs. However, those supervised learning models that are trained by fixed‐size patches generally lack compatibility and efficiency when local regions display a range of structural variations, which may lead to overfitting issues and the consumption of computing resources [170]. Inspired by the natural similarity between crystal (atoms and bonds) and graph data (nodes and edges), Wang et al. described a few‐shot learning framework based on an equivariant graph neural network (EGNN, Figure 11b(i)) to analyze a library of atomic structures (e.g., vacancies, phase boundaries, grain boundaries, doping, etc.), showing significantly promoted accuracy and reduced computing parameters compared to the image‐driven DL models, which is especially evident for those aggregated vacancy lines with flexible lattice distortion (Figure 11b(ii)) [171]. Furthermore, a series of composite defects was efficiently identified through a task chain assembled by the historically trained EGNN sub‐models for every single atomic structure.

To break the limitations of supervised modeling that only enable the analysis of defect configurations within the training datasets, Dan et al. developed an unsupervised learning framework based on Zernike Polynomials (ZP) and force‐relaxed clustering to explore the structural diversity of defective TMDs without upfront intervention (Figure 11c). ZP constitutes a comprehensive set of orthogonal basis functions capable of decomposing image patches into a linear combination of polynomials, each carrying specific physical significance related to astigmatisms. Therefore, the spatial context of the defect configurations can be effectively modeled from the fitted Zernike coefficients with good interpretability. After clustering the extracted Zernike coefficients within the feature space, a library of defect configurations, including Fe‐dopants, vacancies, and stacking faults with continuously varied patterns, etc., has been efficiently analyzed in defective WS_2_ monolayer and MoS_2_ bilayer. Also, the generability of Zernike features has been demonstrated across a wider range of TMD systems, with which a novel defect configuration referred to as antisite Te adatom (Te_ads‐Mo_) was discovered in a defective MoS_2−x_Te_x_ alloys containing thousands of atoms [172].

### 4.3 ML for New Knowledge Inspiration

**FIGURE 12:**
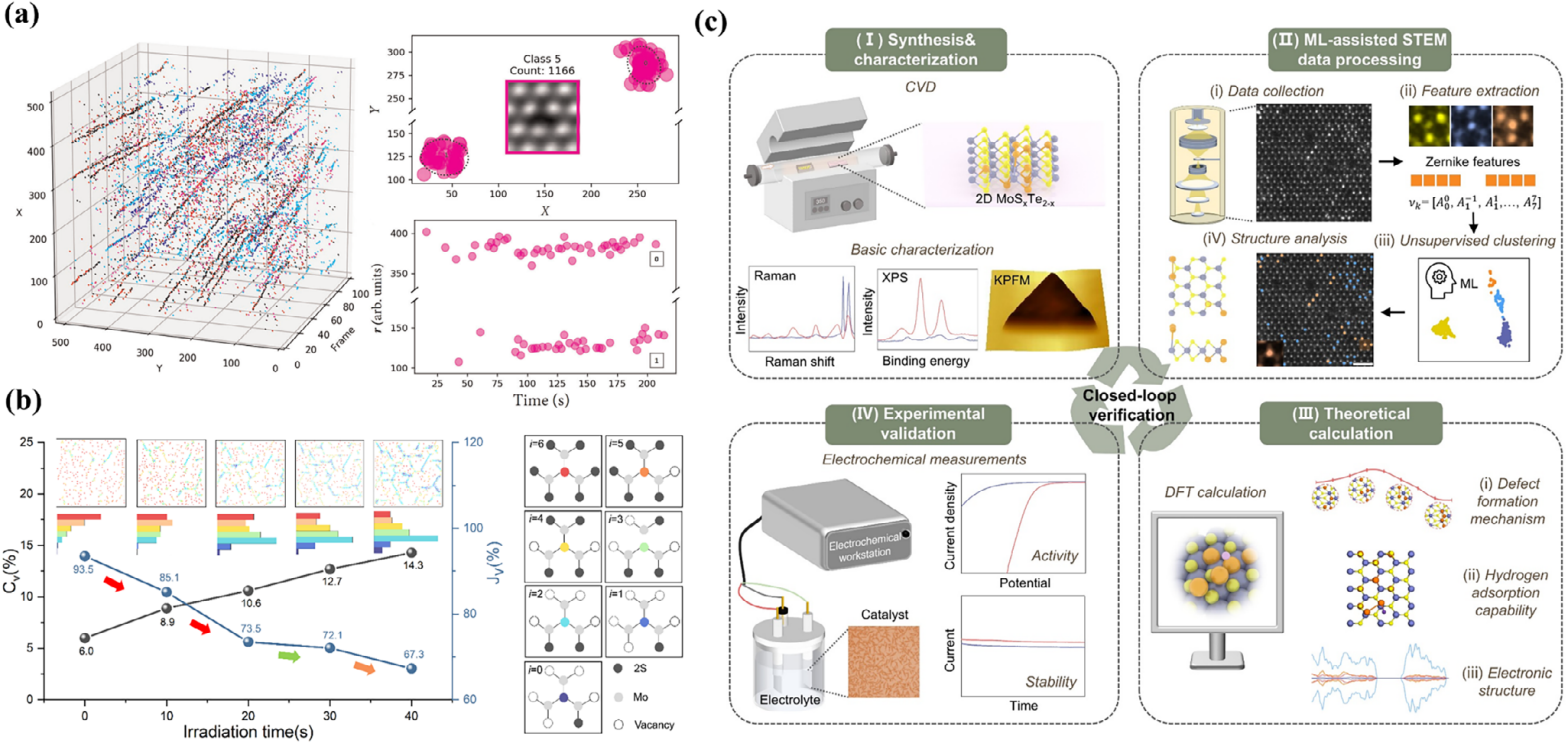
Typical examples of ML‐empowered TEM analytics for new knowledge inspiration. (a) Spatiotemporal trajectories of the detected defects and the analysis of diffusion behavior for a selected defect structure [174]. Copyright 2019, Springer. (b) Plots showing the vacancy concentration (*C_v_*) and the alloying degree of S vacancies (*J*_v_) as a function of irradiation time. Copyright 2025, Wiley‐VCH [171]. (c) Schematic showing the experimentally validated ML‐driven paradigm for high‐efficiency catalyst design [172]. Copyright 2025, Wiley‐VCH.

Assisted by the well‐designed ML models, diversified defects nowadays can be automatically and accurately recognized for statistical investigation (Table 2). However, an intriguing topic beyond visualization raises: “What can we do with this information?” To answer this question, Ziatdinov et al. provide a spatiotemporal diagram of the defect trajectories (Figure 12a) according to the statistical identification results from the time series STEM images of Mo‐doped WS_2_ under continuous electron beam irradiation [174]. Based on the 2D projection from the 3D spatiotemporal diagram, the diffusion coefficients of different defects can be precisely calculated by extracting the coordinates of each selected “defect flow.” Also, the dynamics of different defects can be traced, where the defects associated with S and W (blue and pink dots in Figure 12a) were found to form shorter trajectories compared to Mo‐based defects (black and red dots in Figure 12a), which can be attributed to the formation of W and S species from the extended clusters of the deposited WS_2_ materials that fill these vacancies. Assisted by the ML‐assisted analytics, the beam‐induced material degradation process can be mapped at the atomic level to provide insight into point defect dynamics and reactions. Besides, benefiting from the inherent intuitiveness of graph representation, Wang et al. revealed the self‐assembly behavior of SVLs under continuous electron beam irradiation via directly extracting the structure‐related parameters (e.g., defect concentration, local coordination number, atomic‐scale mixing state, etc.) from the HR‐TEM image series involving ∼27 000 atoms. According to the outputs from EGNN model (Figure 12b), the concentration of vacancies (*C_v_*) was found to exhibit a linear increase as the irradiation time prolongs, while the alloying degree (*J_v_*) calculated from the coordination numbers of each vacancy emerge a distinct platform [171], which was further verified to be the broadening growth behavior of the as‐grown long and narrow vacancy lines along the armchair lattice directions. The unclosed anisotropic self‐assembly behavior of SVLs provides guidelines for the precise defect engineering and electronic band modulation at the atomic level for advanced nanodevice design.

In order to bridge the gap between theoretical study and practical validation, Xue et al. further provided an experimentally validated ML‐driven paradigm for high‐efficiency catalyst design, which has a closed research loop involving catalyst synthesis, ML‐assisted atomic defect discovery, theoretical prediction of defect formation and properties, and electrochemical performance validation (Figure 12c) [172]. Based on the newly discovered Te_ads‐Mo_ defect configurations from Zernike feature and uniform manifold approximation and projection (UMAP)‐assisted clustering, the formation mechanism, theoretical properties, and the catalytic performance of these defects were systematically investigated via DFT calculations and electrochemical experiments, showcasing how ML and researchers seamlessly cooperate in a scientific workflow for catalyst development. Several works beyond 2D TMDs also give examples to answer the question, like the ML‐assisted analytics of invisible H atoms in structural materials for preventing hydrogen embrittlement [175], and the establishment of a high‐level package workflow for efficient prediction of defect properties in graphene using the ML‐derived atomic models [176].

In short, the ML‐empowered defect analytics has leveraged state‐of‐the‐art ML methodologies to enable new capabilities for retrieving statistical information from TEM images with high accuracy and efficiency. However, due to the structural diversity of defects and the unavoidable instrumental deviations, even small local perturbations may cause significant errors in the predicted outcomes. Since ML algorithms typically process data in a black‐box manner without any physical constraints, it is crucial to comprehend the operational characteristics that underlie the outputs and provide suitable constraints (such as physical information or prior knowledge) to enhance both accuracy and versatility of the models [177]. In addition, research in ML‐empowered defect analytics is still in its early stages, which lacks strong connections with the front‐end defect design and the back‐end defect synthesis. A full‐chain support based on ML‐empowered defect analytics is therefore highly expected to realize intelligent and efficient defect engineering in TMDs for energy storage applications.

## 5 Summaries and Future Perspectives

This review systematically summarizes typical atomic defects in TMDs, as well as their effects on optimizing electrochemical redox kinetics and stability. Additionally, recent advances in defect engineering of layered TMDs for their applications as electrode materials, catalysts, electrolyte additives, and artificial interlayers in various types of electrochemical energy storage devices, including batteries and supercapacitors, have been elaborated. Given the indispensability of TEM in revealing the structure–property correlation across diversified defect configurations, we discussed the major challenges in atomic structure analytics in terms of accuracy and efficiency, and reviewed the progress of ML‐based methodologies for high‐throughput TEM data processing. Benefiting from the emerging ML algorithms, high‐quality data settings, atomic column detection, and defect analysis, can be realized to unveil statistically grounded information from a large corpus of TEM data for new structure and knowledge discovery. Although defective TMDs and ML‐empowered TEM data analytics have made remarkable advancements in sustainable energy storage systems, there remain several challenges worth being studied and solved.

**FIGURE 13:**
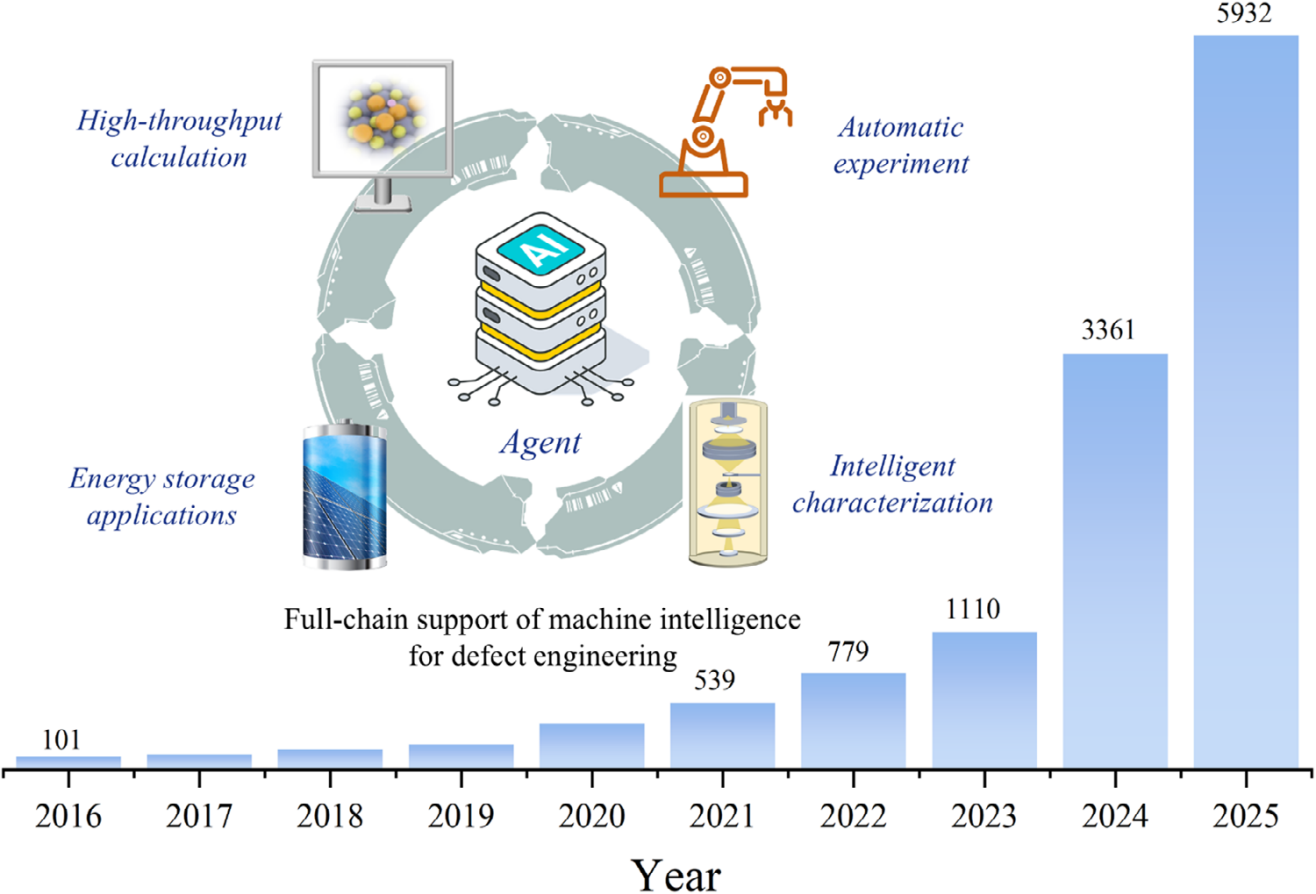
Publications about “AI for Materials” over the past decade, and the inset image showing a schematic of full‐chain support of machine intelligence for defect engineering. The data are acquired from the Web of Science using “AI for Materials” as the keyword.

(1) The gap between laboratory research and scalable application needs to be bridged. Although defective TMDs have demonstrated optimized electrochemical performance when employed in sustainable energy storage devices, several challenges still need to be addressed before practical applications. At first, most defective TMDs have only been tested with limited active material loading (generally below 2 mg·cm^2^) and surplus electrolyte in half cells, but their cycle stability remains unclear when assembled into high‐energy‐density‐packed full cells. Second, the existing synthesis methods, like CVD, hydrothermal, and solvothermal processes, lack efficiency and consistency when extending to scale‐up experiments. In addition, most of the defective TMDs are designed as porous structures to improve the electrochemical reaction activity, but the concurrent decrease in tap density will compromise the electrode manufacturability. To merge the gap between the laboratory research and scalable application, on the one hand, standardized test protocols, like specified areal capacity, electrolyte amounts, and negative/positive ratio, should be established to improve the reliability of the tests; on the other hand, novel structure design, like the hierarchical structures [178], and fabrication strategies, like the “integrated” blowing strategy that facilitates the direct synthesis of free‐standing TMDs‐based electrode [179], need to be developed to promote the scalability, consistency, and manufacturability. Furthermore, sustainable raw materials and novel 2D materials beyond TMDs [180, 181, 182, 183] should be considered as viable alternatives for reducing production costs and implementing economically feasible processes.

(2) The dynamics of defects upon the electrochemical redox process require in‐depth investigation. Although a library of defects has been extensively analyzed via static or *ex situ* TEM, defects in TMDs will concurrently evolve alongside gradual shifts in physicochemical environments as the energy devices operate over an extended time [184]. To spatiotemporally monitor defect evolution under operating conditions, the existing in situ TEM techniques that incorporate electrochemical cells within the chamber should be fully exploited for real‐time observation of the defect evolution dynamics.^[^ ^185^ ^]^ Also, multimodal approaches that seamlessly integrate in situ TEM techniques with electron energy loss spectroscopy, X‐ray diffraction, Raman, etc., and in combination with computational modeling, are highly expected for a more comprehensive understanding of the atomic structure of defects in TMDs.

(3) In terms of ML‐driven microscopy analysis, the current data‐driven methodologies are only capable of performing specific defect analysis tasks within a limited scope, thus lacking adaptivity to the real TEM analysis scenarios featuring diversified atomic configurations, scales, and imaging conditions. To promote the universality of ML models, physics‐informed ML approaches that incorporate domain knowledge and physical laws from crystallography, material science, and electron microscopy, etc., should be well established to improve the training and inference efficiency of the models toward few‐shot or even zero‐shot learning [156]. Moreover, joint efforts from both electron microscopy and computer science communities should be made to establish a versatile platform with continuous learning capabilities, which enables deep engagement with the current TEM systems to execute new tasks across broad material systems and imaging conditions with real‐time feedback.

(4) The front‐end design and synthesis of defective TMDs still strongly rely on the traditional trial‐and‐error method, which suffers from low efficiency, high cost, and poor transferability. Recently, the emerging interests of “AI for Materials” that involves large language models, high‐throughput theoretical calculation, and robotic automation has demonstrated their remarkable capability in property predictions, high‐throughput experiments, autonomous decision‐making, and process optimization across different sections of material innovations [186, 187, 188, 189], bringing great opportunities for the development of a full‐chain support toward intelligent and efficient defect engineering in energy storage devices (Figure 13).

In all, defective TMDs hold great promise for applications in sustainable energy storage devices, and the emerging field of ML has been transforming the research paradigm, encompassing not only defect characterization but also the entire chain of material innovations. Despite remaining challenges, the deep integration of ML with materials science holds boundless potential for precise and efficient defect engineering in TMDs in the foreseeable future.

## Conflicts of Interest

The authors declare no conflicts of interest.

## Data Availbility Statement

The authors have nothing to report.
